# Application of a comprehensive approach to pathogen screening in a stowaway rat on an airplane

**DOI:** 10.1038/s41598-025-13199-6

**Published:** 2025-08-30

**Authors:** Elisa Heuser, Arnt Ebinger, Silva Holtfreter, Silver A. Wolf, Andreas E. Zautner, René Ryll, Stephan Drewes, Beate Matzkeit, Bernd Hoffmann, Dirk Höper, Markus Keller, Allison Groseth, Gottfried Wilharm, Daniel M. Mrochen, Anna Obiegala, Frank Doss, Calvin Mehl, Tobias Eisenberg, Sandra Niendorf, Sindy Böttcher, Axel Karger, Charlotte Schröder, Eric Ehrke-Schulz, Katja Schmidt, Martin Beer, Martin H. Groschup, Torsten Semmler, Gerald Heckel, Martin Pfeffer, Claudia Wylezich, Rainer G. Ulrich

**Affiliations:** 1 Institute of Novel and Emerging Infectious Diseases, Friedrich-Loeffler-Institut, Federal Research Institute for Animal Health, Südufer 10, 17493 Greifswald-Insel Riems, Germany; 2 Institute of Diagnostic Virology, Friedrich-Loeffler-Institut, Federal Research Institute for Animal Health, Südufer 10, 17493 Greifswald-Insel Riems, Germany; 3 Institute of Immunology, University Medicine Greifswald, 17475 Greifswald, Germany; 4 Genome Competence Centre (MF1), Robert Koch Institute, Nordufer 20, 13353 Berlin, Germany; 5 Institut für Medizinische Mikrobiologie und Virologie, Universitätsmedizin Göttingen (UG), Kreuzbergring 57, 37075 Göttingen, Germany; 6 Institute of Molecular Virology and Cell Biology, Friedrich-Loeffler-Institut, Federal Research Institute for Animal Health, Südufer 10, 17493 Greifswald-Insel Riems, Germany; 7 Wernigerode Branch, Robert Koch Institute, Burgstr. 37, 38855 Wernigerode, Germany; 8 Institute of Animal Hygiene and Veterinary Public Health, University of Leipzig, An den Tierkliniken 1, 04103 Leipzig, Germany; 9 Clean Frank Doss GmbH, Kampstraße 27, 16792 Zehdenick, Germany; 10 Landesbetrieb Hessisches Landeslabor (LHL), Schubertstraße 60 - Haus 13, 35392 Gießen, Germany; 11 Department of Viral Gastroenteritis and Hepatitis Pathogens and Enteroviruses, Robert Koch Institute, Seestraße 10, 13353 Berlin, Germany; 12 Regional Reference Laboratory of the WHO/Europe for Poliomyelitis, Robert Koch Institute, Seestraße 10, 13353 Berlin, Germany; 13 Department of Experimental Animal Facilities and Biorisk Management, Friedrich-Loeffler-Institut, Federal Research Institute for Animal Health, Südufer 10, 17493 Greifswald- Insel Riems, Germany; 14 Lehrstuhl für Virologie und Mikrobiologie, Zentrum für biomedizinische Ausbildung und Forschung (ZBAF), Universität Witten/Herdecke, Stockumer Strasse 10, 58453 Witten, Germany; 15 Microbiological Diagnostics, German Cancer Research Center Heidelberg, Im Neuenheimer Feld 280, 69120 Heidelberg, Germany; 16 Institute of Ecology and Evolution, University of Bern, Baltzerstrasse 6, Bern, 3012 Switzerland; 17 German Centre for Infection Research (DZIF), Partner Site Hamburg-Lübeck-Borstel-Riems, Greifswald-Insel Riems, Germany; 18 Present Address: Institut für Medizinische Mikrobiologie und Krankenhaushygiene, Universitätsklinikum Magdeburg A. ö. R, Leipziger Str. 44, 39120 Magdeburg, Germany

**Keywords:** High-throughput sequencing, Picobirnavirus, *Staphylococcus aureus*, Commensals, Multiplex serological assay, Pathogen importation, Ecology, Microbiology, Zoology, Diseases, Risk factors

## Abstract

In April 2017, a rat was observed on an airplane during a flight from Miami (USA) to Berlin (Germany). After landing in Berlin, significant efforts were made to trap the rat and disinfect the airplane. As rats are known reservoir hosts for a variety of zoonotic pathogens, this event necessitated the establishment of a standard workflow for the detection of rodent-borne pathogens. Tissue and blood samples were collected to screen for zoonotic pathogens and other known and novel infectious agents using an array of open-view methods (cultivation and characterization of bacteria, high-throughput sequencing) and pathogen-specific methods (e.g. PCR, RT-PCR and multiplex serology). The black rat (*Rattus rattus*), as confirmed by mtDNA sequences, carried several infectious agents. Cultivation experiments revealed the presence of seven bacterial and two fungal genera. In addition, a methicillin-susceptible *Staphylococcus aureus* strain of MLST-CC45 was detected by culture-based approaches, and its full genome was sequenced. High-throughput sequencing identified novel picobirnaviruses and various bacterial genera, the majority of which represent commensals rather than pathogens. Despite the diversity of bacterial, viral, and fungal species that can be expected in wild rats, only a few zoonotic and non-zoonotic pathogens were detected in the stowaway rat. Nonetheless, this incident highlights the potential of international (and cross-continental) dissemination of pathogens and the need for a standardized workflow to provide comprehensive coverage of the diversity of microorganisms in such animals.

**Supplementary Information:**

The online version contains supplementary material available at 10.1038/s41598-025-13199-6.

## Introduction

Commensal rodents, such as Norway or brown rats (*Rattus norvegicus*), black, roof or “ship” rats (*Rattus rattus*) and house mice (*Mus musculus*), are ubiquitous urban pests, with Norway rats and house mice also often used as pet, feeder or laboratory animals^1–3^. Their close association with humans is reflected in the evolutionary history and dispersal of these species^4–6^. Although their historical anthropogenic dissemination is well documented, little is known about the current global transport of rodents via ships, planes, trains and motor vehicles. Despite significant pest rodent management efforts around the globe, phylogenetic analyses of globally distributed pathogens suggest an ongoing worldwide transport of rodents^7^.

Rats, and Norway rats in particular, are well-known reservoirs of zoonotic pathogens, e.g. *Leptospira interrogans*, *Streptobacillus moniliformis*, and Seoul orthohantavirus (SEOV; species *Orthohantavirus seoulense*)^7–9^. Although rat hepatitis E virus (ratHEV; species *Rocahepevirus ratti*) was discovered more than a decade ago^10^, its zoonotic potential has only recently been confirmed^11–13^. Increased screening efforts have also identified several rat-specific infectious agents such as polyoma-, papilloma- and herpesviruses^14–16^. However, high-throughput sequencing (HTS) approaches revealed a large number of viruses that were detected in both commensal rats and house mice^17–19^. In addition, rats may play a role in the dissemination of pathogens, such as noro-, entero- and astroviruses and extended beta-lactamase-producing Enterobacterales^20,21^. Rats were also reported to be colonized by strains of methicillin-resistant *Staphylococcus* (*S.*) *aureus* (MRSA)^22^. A comparative study of nasal *S. aureus* isolates revealed a natural occurrence of *S. aureus* in wild rats, as well as colonization in wild and laboratory rats by exposure to livestock- and human-associated *S. aureus* strains, respectively^22^.

While half a century ago, intercontinental transport of rats was only facilitated by ships, nowadays the increase in global air traffic further facilitates unintentional introduction of animals from other countries^23,24^. Pests are strictly controlled for on air traffic to prevent incursion into novel regions and to prevent damage to on-board electronics as well as for hygiene and infection control reasons. As commensal rodents are known to harbor a diversity of important pathogens, and are well adapted to urban environments, their unintentional transport could have significant implications for human and animal health. Here, we describe the fumigation of an airplane, including localization of a stowaway rat that was found during a flight from the USA to Germany, but might have been on board from the beginning (Dubai, United Arab Emirates (UAE)), its species identification as well as a comprehensive pathogen screening, including viruses, bacteria, parasites and fungi. The reported workflow employed various untargeted open-view, multiplex and target-specific methods and whole-genome sequencing.

## Methods

### Sample collection, dissection and species identification

All methods were carried out in accordance with relevant national and international guidelines and regulations. The removal of the stowaway rat from the airplane follows the regulations given by the International Air Transport Association (IATA) and the World Health Organization (WHO; World Health Organization 2015). According to the American Veterinary Medical Association (AVMA), Guidelines for the Euthanasia of Animals (2020), the employed euthanasia method for the stowaway rat is consistent with the commonly accepted norms of veterinary best practice.

On March 24 in 2017, a rodent was spotted on an airplane from Miami (Florida, USA) to Berlin (Germany) that started its journey in Dubai (UAE). In accordance with international regulations, the aviation authority grounded the airplane at Berlin-Tegel airport. After internal fumigation of the whole airplane with CO_2_ gas, the rat (H17/01) was located (with the help of a hunting dog) and collected for further investigations. The rodent capture and subsequent investigation workflows are shown in Figure S1.

The animal was frozen and sent to the Friedrich-Loeffler-Institut (FLI), Greifswald-Insel Riems, for necropsy and coordination of pathogen screening. The frozen carcass was thawed at 4 °C and dissected in a biosafety level 3 containment laboratory, with corresponding hygiene measures for the personnel, following a standard protocol, i.e. samples were taken from the heart, lung, chest cavity fluid (CCF), brain, liver, spleen, kidney, trachea, tongue, nose, ear, intestine, feces and the tail tip, then stored at -20 °C. Weight, sex, body and tail lengths were documented during dissection.

The rat species was determined by cytochrome *b* gene sequencing, as previously described^25^. Additionally, the entire cytochrome *b* gene and the whole mitochondrial genome of the rat was extracted from HTS datasets via reference mapping and phylogenetically analyzed (see below).

### Microbial isolation, cultivation and pathogen characterization

To demonstrate reproducibility, two cultivation trials were performed for bacterial and fungal organisms at different institutions (FLI and University Medical Center Göttingen (UMG)). In a first trial, cultivation of fecal samples was initiated with a bacteriological plate set consisting of cattle blood Columbia agar plates (Sifin, Berlin, Germany) under aerobic and anaerobic incubation conditions, as well as an aerobic incubation on a Gassner plate (Water blue Metachrome yellow agar according to Gassner, modified, Sifin), all at 37 °C. For *Salmonella* exclusion diagnostics, Rappaport medium (Rappaport-Vassiliadis soy peptone (RVS) broth, Oxoid, Wesel, Germany), and an XLD (xylose lysin desoxycholat agar (XLD agar), Carl Roth, Karlsruhe, Germany) plate were used.

In a second trial, rat fecal samples were incubated in three different atmospheres: (I) Aerobic conditions, at 37 °C: Columbia agar supplemented with 5% sheep blood (Becton Dickinson GmbH, Heidelberg, Germany), *Salmonella Shigella* agar (Becton Dickinson GmbH), MacConkey agar no. 3 (Thermo Fisher Scientific Inc., Waltham, MA, USA), Sabouraud dextrose agar (Thermo Fisher Scientific Inc.), Chapman/Mannitol salt agar (Becton Dickinson GmbH), and *Yersinia* selective agar (Becton Dickinson GmbH), (II) Microaerophilic (CampyGen sachets, Thermo Scientific Oxoid) or capnophilic (BD BBL™ CO_2_ generators, Becton Dickinson GmbH) conditions at 37 °C: Campylosel agar (bioMérieux, Nürtingen, Germany) and Mueller Hinton Chocolate agar (Becton Dickinson GmbH), (III) Anaerobic conditions (BD BBL GasPak™ Plus, Becton Dickinson GmbH) for 2 to 14 days at 37 °C: chromID™ *C. difficile* agar (bioMérieux, Nürtingen, Germany), Schaedler agar with vitamin K1 and 5% sheep blood (Becton Dickinson GmbH), and Schaedler kanamycin-vancomycin agar with 5% sheep blood (Becton Dickinson GmbH).

For detection of *Acinetobacter baumannii*, tracheal sample material was suspended in 3 mL of mineral medium^26^ supplemented with 0.1% acetate as the sole source of carbon and energy, and incubated at 37 °C with constant shaking^27^. After zero, five, and 24 h, respectively, 100 µL of the suspension were spread onto *Acinetobacter* selective medium (CHROMagar, La Plaine St-Denis, France; without CHROMagar multidrug-resistant supplement) and incubated for 24 h at 37 °C.

### Isolation and characterization of *Staphylococcus aureus*

**Fig. 1:**
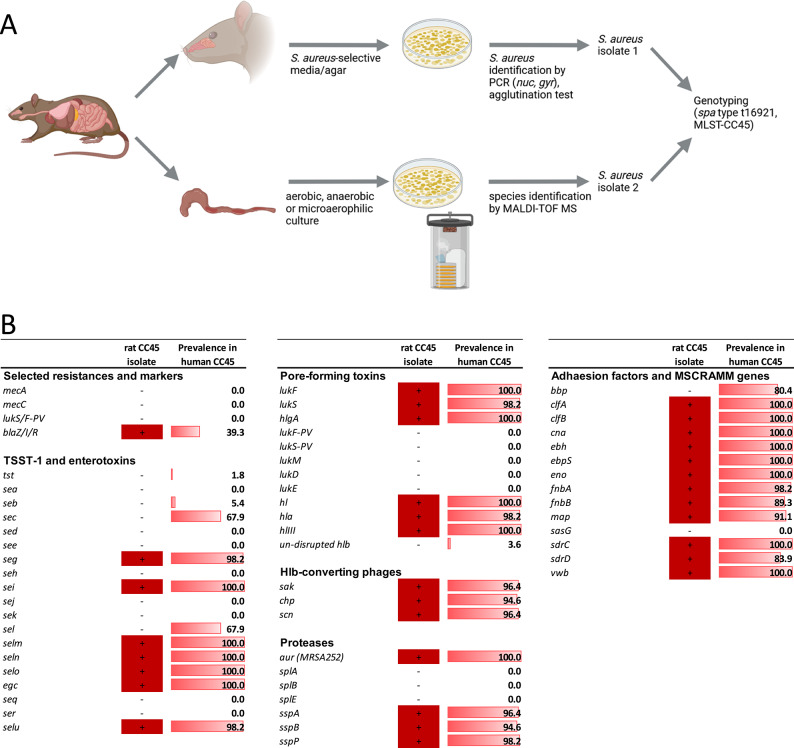
Nasal and gastrointestinal colonization of the airplane rat with a CC45-MSSA strain. *Staphylococcus* (*S*.) *aureus* was isolated from the nose using selective agars and media, and identified by an *S. aureus-*specific latex agglutination test as well as an *S. aureus*-specific multiplex PCR. Colon content was streaked onto a set of different selective media including Chapman mannitol salt agar on which a second *S. aureus* isolate could be cultured. Microbial species identification by MALDI-TOF MS was performed using a Bruker Autoflex III system. *S. aureus* isolates from nose and colon both belonged to *spa* type t16921 (CC45) (**A**). Staphylococcal resistance, virulence and immune evasion genes were detected with a commercial DNA array. The presence of CC45-associated genes in the nasal rat CC45-MSSA isolate vs. 56 human CC45-MSSA isolates is depicted (**B**).

As illustrated in Fig. 1, *S.* *aureus* was isolated from the homogenized rat nose in a culture-based approach, as previously reported^28^. Furthermore, *S. aureus* was isolated from rat intestine content (feces) by streaking a pea-sized amount of the feces sample onto a Columbia sheep blood agar plate using the quadrant streaking technique. The plate was then incubated overnight under aerobic conditions at 37 °C.

*S. aureus* identity was confirmed with a colony multiplex-PCR for the *S. aureus* gyrase gene and an *S. aureus-*specific latex agglutination test (Staph Xtra Latex kit, ProLex, Richmond Hill, ON, Canada), as previously reported^28^.

*Spa* genotyping and multilocus sequence typing (MLST) were performed as previously described^29,30^. Virulence and resistance genes were detected with the StaphID DNA microarray (Alere Technologies GmbH, Jena, Germany)^28,31^. The *mecC* gene encoding a methicillin resistance gene product was detected by PCR, as previously described^32^. Human nasal clonal complex (CC) 45 isolates (*n* = 56), which were obtained in the population-based study SHIP-TREND-1 between 2016 and 2019^33^ (approval number BB 39/08 by the Ethics Committee at the University Medicine Greifswald, Germany) by culture-based approaches as previously reported^34^, were used for the comparison of samples in our study.

### Whole-genome sequencing, genome reconstruction and annotation, and phylogenetic analysis of *Staphylococcus aureus* isolates

Whole-genome sequencing (WGS) was conducted following an established protocol^35^. In brief, genomic DNA was extracted from both cecum and nasal isolates, and subjected to initial paired-end (2 × 150 base pairs, bp) next generation sequencing using an Illumina MiSeq platform. Long-read sequencing was subsequently performed on the Oxford Nanopore Technologies (ONT) platform and combined with the Illumina data for genomic reconstruction. For this, long-read sequences were assembled using the flye software (v2.9.2-b1786)^36^. Next, short-read polishing of the assemblies was performed via the HyPo package (v1.0.3; https://github.com/kensung-lab/hypo). Species-specific annotation was subsequently conducted through Bakta (v1.10.0)^37^. MLST was performed using mlst (v2.23.0) (https://github.com/tseemann/mlst). Circular genomes were visualized using GenoVi (v0.4.3)^38^. Single nucleotide polymorphisms (SNP) between both isolates were assessed using snippy (v4.6.0) (https://github.com/tseemann/snippy). Plasmid sequences were further investigated via PlasmidFinder (v2.1)^39^.

In order to assess the phylogenetic relationship of the isolates within the context of publicly available data, a total of 1,689 genomes assigned to sequence type 45 (ST45) were retrieved from Pathogenwatch (dated 2024-11-16) (https://pathogen.watch/). When available, metadata regarding geographical and temporal distribution were also included. The genome collection was annotated analogously to the sequenced isolates, and both were utilized for further population study. The population structure of the sample collection was investigated through phylogenetic reconstruction of a maximum-likelihood tree. A gene-by-gene approach was selected to first establish a shared set of conserved (“core”) genes based on the annotated bacterial assemblies in combination with the roary software (v3.13.0)^40^. Using this approach, a total of 1,528 conserved genes were identified, which were present in at least 99% of the strains (protein sequence similarity ≥ 95%, sequence coverage ≥ 90%). This was followed by gene-wise alignments using the MAFFT software (v7.520)^41^, and subsequent concatenation of the alleles per sample. The resulting alignment was then utilized to infer a phylogeny through RAxML-NG (v1.2.0)^42^ using a General Time Reversible (GTR) model and gamma correction for rate variation among sites. Finally, iTOL (v6.8.1)^43^ was used to visualize the isolates within the context of the available genomes of the corresponding CC. Average nucleotide identity (ANI) values were computed between the isolates and the genome collection through fastANI (v1.34)^44^. The presence of *mec*A was assessed through ABRicate (v1.0.1) (https://github.com/tseemann/abricate) using the National Center for Biotechnology Information (NCBI) antimicrobial resistance (AMR) database (dated 2025-01-14) with default settings (minimum coverage and identity ≥ 80%).

### Nucleic acid extraction and RT-PCR and PCR investigations

For pathogen specific nucleic acid detection, standard protocols for RNA and DNA extraction were used (Table S1). The extracted nucleic acids were stored at − 80 °C until further use.

Generic and pathogen-specific conventional and real-time RT-PCR and PCR investigations for 20 viruses, ten bacteria and one endoparasite followed established standard protocols (Table S1). For the detection of polyoma and papilloma viruses, enrichment of potential circular viral DNA, in the background of linear host DNA, was performed via rolling circle amplification (RCA) using the TempliPhi RCA kit (GE Healthcare, Piscataway, NJ, USA), as previously described^45^.

The species identification of the yeast, *Cutaneotrichosporon mucoides*, was conducted via 18S ribosomal RNA (rRNA) gene sequencing using a previously published protocol^46^.

### Matrix-assisted laser desorption/ionisation time-of-flight mass spectrometry (MALDI-TOF MS) identification of bacterial and fungal species

The species of morphologically distinct colonies were identified using the Bruker Biotyper MALDI-TOF MS system (Bruker Daltonics GmbH & Co. KG, Bremen, Germany) using the ethanol/formic acid extraction method following the recommended standard procedure^47^. Thereafter, one µL of the supernatant was spotted onto polished steel MALDI target plates. The air-dried whole cell extracts were overlaid with 2 µL of a saturated solution of α-cyano-4-hydroxycinnamic acid in 50% acetonitrile / 2.5% trifluoroacetic acid and dried again at ambient temperature. Spectra were acquired with an Autoflex III and Ultraflextreme mass spectrometer (Bruker) in the linear positive mode, in the mass range of 2,000–20,000 Da. The instrument was externally calibrated in the mass range between 3,637.8 and 16,952.3 Da using the Bacterial Test Standard (BTS) calibrant (Bruker Daltonics, Bremen, Germany) before measurement. Samples were identified using the MALDI Biotyper software (version 3.1) together with the Bruker reference library (database release 2017). Results with MALDI Biotyper identification scores greater than 2.000 were deemed sufficient for species identification^48–51^. For the mass spectrometric species identification of fungi, the established method for bacteria was expanded through an extended direct transfer (eDT) approach. In this process, after applying the sample to the MALDI target plate, an on-target lysis (OTL) was performed using one µL of 70% formic acid. Following the evaporation of the formic acid, the matrix solution was applied.

### Metagenomics analyses

Ion Torrent S5 compatible libraries of liver (L2208), spleen (L2209), lung (L2210), feces (L2211), CCF (L2212), brain (L2213), and kidney (L2214) were prepared according to Wylezich et al. 2018^52^. Sequencing was performed on an Ion Torrent platform (either PGM or S5XL) using a suitable chip with a mean read length of 400 bp. Metagenomic analyses were conducted by the use of the RIEMS pipeline^53^. In addition to RIEMS, DIAMOND was used to screen for additional picobirnavirus (PBV) hits^54^. The PBV consensus sequences were determined by an iterative assembly and mapping approach through the Genome Sequencer software suite (v3.0; Roche), after extraction of the PBV reads from the datasets using RIEMS and DIAMOND. Nucleotide sequences were in-silico translated into protein sequences with EMBOSS version 6.3.1^55^. For comparison, two metagenome datasets originating from Norway rat feces in Berlin, Germany, from the Sequence Read Archive (SRR1438008, library Mu/10/1772; SRR1438014, library Mu/10/1805) were used for viral genome assembly, because of the high number of PBV reads identified by Sachsenröder et al. 2014^18^. PBV reads were identified in the datasets using DIAMOND, extracted and assembled using SPAdes (version 3.13.1)^56^.

Prokaryotic and eukaryotic suspected taxa, according to the RIEMS results protocols, were verified via reference mapping (Genome Sequencer software suite, versions 2.6; Roche) using small subunit rRNA sequences as described^57^. Afterwards, datasets were again mapped against the obtained contigs of the nearly complete 16S/18S rRNA gene sequences using different identity thresholds (-mi 95, 98, 100). The following sequences were used as references: the bacteria *Acinetobacter baumannii* strain ATCC 19606^T^ (NR_117620), *Anaplasma phagocytophilum* strain Webster (NR_044762), *Bartonella grahamii* (HG726044), *Bartonella henselae* strain Houston-1 (NR_074335.2), *Borrelia burgdorferi* strain G2 (M60967), *Clostridioides difficile* ATCC 9689/DSM 1296 (NR_112172), *Leptospira ainlahdjerensis* strain 201903070 (NR_181724), *Metamycoplasma/ Mycoplasma arthritidis* strain PG6 ^T^ (M24580.2), *Mycoplasmopsis pulmonis* strain PG34 (NR_041744), *Rickettsia japonica* YH (NR_074459.2), *Rodentibacter pneumotropicus* strain NCTC 8141 (NR_118763), *Streptobacillus moniliformis* strain DSM 12112^T^ (NR_074449), the fungi *Cystobasidium laryngis* (AB126649) and *Trichosporon mucoides* (AB001763.2), and the protists *Babesia microti* strain RI (XR_002459986) and *Goussia bayae* isolate Potomac (MH758783).

### Bioinformatic and phylogenetic analyses

For phylogenetic analysis of PBV sequences, we used the amino acid sequence of the RNA-dependent RNA polymerase (RdRp) encoded by the segment 2 according to Yinda et al. (2018)^58^. Sequence alignments were conducted with MAFFT^59^, as implemented in Geneious Prime 10.2.3 (Biomatters, Auckland, New Zealand). A phylogenetic maximum-likelihood tree was constructed using RAxML 8.2.11^60^, implemented in Geneious Prime 10.2.3 using default settings and 100 bootstrap replications.

The complete mitochondrial genome of the rat was extracted from HTS datasets (L2208, L2210, L2211, L2213, L2214) using NC_012374 (*Rattus rattus*) as reference sequence. The obtained sequence was aligned with mitochondrial sequences of the genus *Rattus*, retrieved from GenBank. A maximum-likelihood phylogenetic tree was constructed using PhyML version 3.0^61^ using the Generalized Time Reversible (GTR) nucleotide substitution model with a gamma distribution and a proportion of invariable sites, and 1,000 bootstrap replications within the Geneious Prime 10.2.3 software package. The best-fit nucleotide substitution model was determined by J Model Test2^62^. In parallel, a Bayesian analysis was performed in MrBayes version 3.2.6^63^ for 50 million generations, sampled every 5,000 generations and the first 25% were discarded as burn-in.

### Multiplex serology

The multiplex serology “rat panel” included rat parvoviruses (Kilham rat virus, Toolan´s H-1 virus and rat minute virus, species *Protoparvovirus rodent 1*; rat parvovirus, species *Protoparvovirus rodent 2*), Sendai virus (species *Respirovirus muris*), rat coronavirus (species *Betacoronavirus muris*), pneumonia virus of mice (species *Murine orthopneumovirus*), mouse adenovirus type 1 (species *Mastadenovirus encephalomyelitidis*), cowpox virus (species *Orthopoxvirus cowpox*), orthohantaviruses including Seoul orthohantavirus (species *Orthohantavirus seoulense*), reovirus type 3 (species *Orthoreovirus mammalis*), rat hepatitis E virus (species *Rocahepevirus ratti*), rat rotavirus (species *Rotavirus betagastroenteritidis*), *Streptobacillus moniliformis*, *Rodentibacter* spp., *Mycoplasma pulmonis* and *Mycoplasma arthritidis*. Except for the detection of antibodies against the bacteria mentioned above, the multiplex serology was based on a glutathione-S-transferase (GST) capture immunosorbent assay in combination with the fluorescent bead technology from Luminex Corp. (Austin, TX, USA). Viral antigens were expressed as GST-tagged fusion proteins and affinity-purified directly on glutathione-casein-coupled polystyrene beads with distinct embedded fluorescent dyes (SeroMap; Luminex Corp.), as previously described^64^. In contrast, bacteria were cultured, lysed and membrane proteins were extracted, before they were directly coupled to polystyrene beads. The general set-up and protocol of the multiplex serology was described by Schmidt et al. 2017^65^. The Luminex analyzer BioPlex200 (BioRad Laboratories GmbH, Munich, Germany) was used to distinguish between the bead sets, and consequently the bound antigen, and to quantify the amount of bound serum antibody using a secondary antibody (biotinylated goat anti-rat IgM/IgG, Jackson ImmunoResearch Laboratories, Inc., West Grove, PA, USA; diluted 1:1,000) and a fluorescent reporter conjugate (streptavidin-R-phycoerythrin). Final antigen-specific median fluorescence intensity (MFI) values were measured for at least 75 beads per bead set, and sample and net values were calculated by subtracting the individual bead background values resulting from a serum-free reaction and from a bead-set loaded with GST-tag only. Samples were defined as positive if the net MFI values were above the calculated cut-off to achieve 98% specificity for seropositivity to the individual antigens on the basis of the receiver operating characteristics (ROC) during the validation process.

## Results

### Characterization of the stowaway rat

**Fig. 2:**
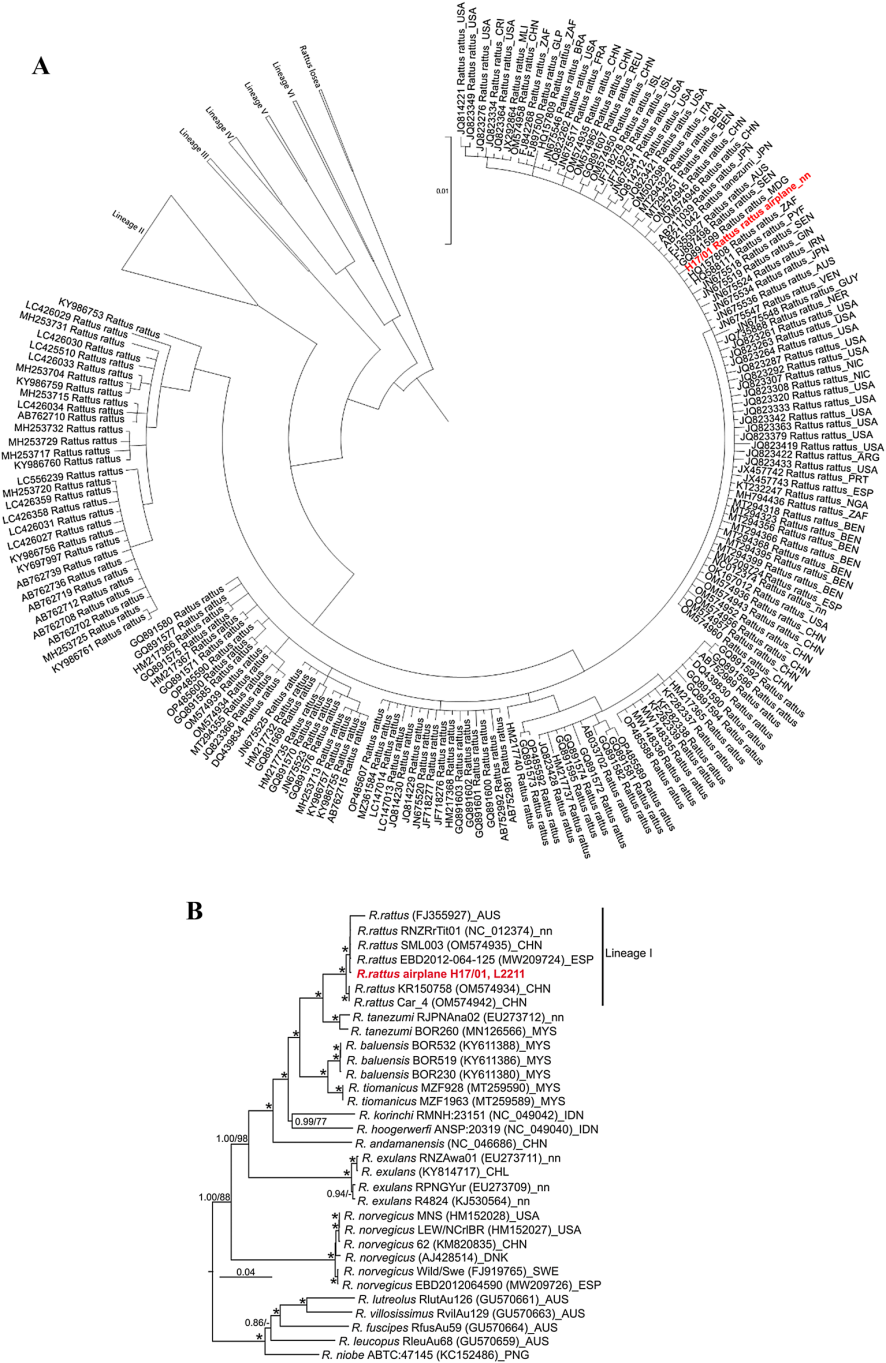
Phylogenetic analysis of complete cytochrome *b* gene (**A**) and complete mtDNA genome (**B**) of the black rat from the airplane (highlighted in red) and representative sequences of black rat lineage I. In panel (**A**), a complete tree of lineage I sequences including the sequence of the rat from the airplane and representative sequences of lineages II – VI is shown; a sequence from *Rattus losea* was used as outgroup. In panel (**B**), complete mtDNA genomes were analyzed in parallel using Bayesian inference (MrBayes, 50 million generations) and maximum-likelihood (PhyML, 1,000 replicates). Where both methods produced congruent nodes, posterior probabilities and bootstraps were provided, i.e. 1.00/100 (as asterisk). Values above 0.8/80 are shown. *Sundamys muelleri* (KY117585) was used as outgroup taxon (not shown). Country codes (ISO 3166-1 alpha-3): ARE, United Arab Emirates; ARG, Argentina; AUS, Australia; BEN, Benin; BRA, Brazil; CHL, Chile; CHN, China; CRI, Costa Rica; CMR, Cameroon; COD, Congo; DNK, Denmark; ESP, Spain; FRA, France; DEU, Germany; GIN, Guinea; GLP, Guadeloupe; GUY, Guyana; HKG, Hong Kong; HUN, Hungary; IDN, Indonesia; IRN, Iran; ISL, Iceland; ITA, Italy; JPN, Japan; MDG, Madagascar; MLI, Mali; MYS, Malaysia; KNA, St. Kitts and Nevis; NER, Niger; NGA, Nigeria; NIC, Nicaragua; PNG, Papua New Guinea; PRT, Portugal; PYF, French Polynesia; REU, Réunion; SEN, Senegal; SVN, Slovenia; SWE, Sweden; USA, United States of America; VEN, Venezuela; ZAF, South Africa; nn, not known.

Phylogenetic analysis of the complete cytochrome *b* gene (1,143 bp) resulted in the species identification of the animal in question as a black rat (*Rattus rattus*), and its classification to the black rat lineage 1, which is distributed worldwide (Fig. 2A). A phylogenetic analysis with the complete mitochondrial genome (16,302 bp) of the rat supports this assignment (Fig. 2B).

### Open view method-based screening for potential pathogens

The pathogen screening was based on a parallel microbiological isolation approach, combined with MALDI-TOF MS, and metagenomics analysis with qualitative evaluation of pathogen presence. To corroborate the screening results, additional specific analyses were carried out (see Table 1 and text below).

**Table 1:** Results of the open-view, multiplex and pathogen specific investigations of the rat.

Taxonomic entity	Sample	PCR and RT-PCR (assay specificity)	Cultivation and mass spectrometry	Serology (assay specificity)	Multiplex serology (assay specificity)	HTS
*Togaviridae*	Brain	*neg.* (Chikungunya virus)	n.a.	n.a.	n.a.	n.d.
*Togaviridae*	Brain	*neg.* (Sindbis virus)	n.a.	n.a.	n.a.	n.d.
*Togaviridae*	Brain	*neg.* (Venezuelan equine encephalitis virus )	n.a.	n.a.	n.a.	n.d.
*Togaviridae*	Brain	*neg.* (Western equine encephalitis virus)	n.a.	n.a.	n.a.	n.d.
*Flaviviridae*	Brain	*neg. *(Zikavirus)	n.a.	n.a.	n.a.	n.d.
*Acinetobacter*	CCF	n.a.	n.a.	n.a.	n.a.	*pos.* (*Acinetobacter* sp.)
*Mycoplasmopsis*	CCF	n.a.	n.a.	n.a.	*neg.* (*M. pulmonis*)	n.a.
*Metamycoplasma*	CCF	n.a.	n.a.	n.a.	*neg.* (*Metamycoplasma arthritidis*)	n.a.
*Rodentibacter*	CCF	n.a.	n.a.	n.a.	*neg.* (*Rodentibacter* sp.)	n.a.
*Streptobacillus*	CCF	n.a.	n.a.	n.a.	*neg.* (*Streptobacillus moniliformis*)	n.a.
*Streptococcus*	CCF	n.a.	n.a.	n.a.	n.a.	*pos.* (*Streptococcus* sp.)
*Adenoviridae*	CCF	n.a.	n.a.	n.a.	*neg.* (Murine mastadenovirus A)	n.a.
*Arenaviridae*	CCF	*neg.* (Arenavirus)	n.a.	n.a.	n.a.	n.a.
*Coronaviridae*	CCF	n.a.	n.a.	n.a.	*neg.* (Murine coronavirus)	n.a.
*Poxviridae*	CCF	n.a.	n.a.	n.a.	*neg.* (Cowpox virus)	n.a.
*Togaviridae*	CCF	*neg.* (Eastern equine encephalitis virus)	n.a.	*neg.* (Eastern equine encephalitis virus)	n.a.	n.a.
*Parvoviridae*	CCF	n.a.	n.a.	n.a.	*neg.* (Kilham rat virus, Rat minute virus, H-1 virus, Rat parvovirus 1)	n.a.
*Pneumoviridae*	CCF	n.a.	n.a.	n.a.	*neg.* (Murine pneumonia virus)	n.a.
*Hantaviridae*	CCF	n.a.	n.a.	*neg.* (Seoul orthohantavirus)	*neg.* (Orthohantaviruses, Seoul orthohantavirus)	n.a.
*Hepeviridae*	CCF	n.a.	n.a.	*neg.* (Rat hepatitis E virus)	*neg.* (Rat hepatitis E virus)	n.a.
*Spinareoviridae*	CCF	n.a.	n.a.	n.a.	*neg.* (Mammalian orthoreovirus)	n.a.
*Sedoreoviridae*	CCF	n.a.	n.a.	n.a.	*neg.* (Rat rotavirus B)	n.a.
*Paramyxoviridae*	CCF	n.a.	n.a.	n.a.	*neg.* (Sendai virus)	n.a.
*Togaviridae*	CCF	n.a.	n.a.	*neg.* (Venezuelan equine encephalitis virus )	n.a.	n.a.
*Togaviridae*	CCF	n.a.	n.a.	*neg.* (Western equine encephalitis virus)	n.a.	n.a.
*Borrelia*	Ear pinna	*neg.* (*Borrelia* sp.)	n.a.	n.a.	n.a.	n.a.
*Rickettsia*	Ear pinna	*neg.* (*Rickettsia* sp.)	n.a.	n.a.	n.a.	n.a.
*Papillomaviridae*	Ear pinna	*neg.* (Papillomavirus)	n.a.	n.a.	n.a.	n.a.
*Acinetobacter*	Feces	n.a.	n.d. /n.d.	n.a.	n.a.	*pos.* (*Acinetobacter* sp.)
*Cellulosimicrobium*	Feces	n.a.	*pos.* (*Cellulosimicrobium cellulans*)/n.d.	n.a.	n.a.	n.d.
*Clostridioides*	Feces	*neg.* (*Clostridioides difficile*)	n.d./n.d.	n.a.	n.a.	*pos.* (*Clostridioides* sp.)
*Enterobacter*	Feces	n.a.	n.d./*pos.* (*Enterobacter cloacae*)	n.a.	n.a.	n.d.
*Brucella*	Feces	n.a.	n.d./*pos.* (*Brucella tritici*)	n.a.	n.a.	n.d.
*Staphylococcus*	Feces	*neg.* (*Staphylococcus aureus*)	*pos.* (*Staphylococcus aureus*)/*n.d*.	n.a.	n.a.	*pos.* (*Staphylococcus* sp.)
*Adenoviridae*	Feces	*neg.* (Adenovirus)	n.a.	n.a.	n.a.	n.d.
*Astroviridae*	Feces	*neg.* (Astrovirus)	n.a.	n.a.	n.a.	n.d.
*Parvoviridae*	Feces	*neg.* (Bocaparvovirus)	n.a.	n.a.	n.a.	n.d.
*Picornaviridae*	Feces	*neg.* (Enterovirus A)	n.a.	n.a.	n.a.	n.d.
*Caliciviridae*	Feces	*neg.* (Norovirus)	n.a.	n.a.	n.a.	n.d.
*Picobirnaviridae*	Feces	n.a.	n.a.	n.a.	n.a.	*pos.* (Picobirnavirus)
*Sedoreoviridae*	Feces	*neg.* (Rotavirus)	n.a.	n.a.	*neg.* (Rotavirus)	n.d.
*Caliciviridae*	Feces	*neg.* (Sapovirus)	n.a.	n.a.	n.a.	n.d.
*Cutaneotrichosporon*	Feces	n.a.	n.d./*pos.* (*Cutaneotrichosporon mucoides*)	n.a.	n.a.	n.d.
*Rhodotorula*	Feces	n.a.	n.d. /*pos.* (*Rhodotorula mucilaginosa*)	n.a.	n.a.	*pos.* (*Rhodotorula* sp.)
*Cystobasidium*	Feces	n.a.	n.a.	n.a.	n.a.	*pos.* (*Cystobasidium* sp.)
*Klebsiella*	Feces	n.a.	*pos.* (*Enterobacter aerogenes*)/n.d.	n.a.	n.a.	n.d.
*Lactobacillus*	Feces	*neg.* (*Lactobacillus* sp.)	*pos.* (*L. gasseri*)/ *pos.* (*L. johnsonii*)	n.a.	n.a.	*pos.* (*Lactobacillus* sp.)
*Ligilactobacillus*	Feces	n.a.	*pos.* (*Ligilactobacillus murinus*)/n.d.	n.a.	n.a.	*pos.* (*Ligilactobacillus* sp.)
*Leptospira*	Kidney	*neg.* (*Leptospira* sp.)	n.a.	n.a.	n.a.	n.d.
*Arenaviridae*	Liver	*neg.* (Arenavirus)	n.a.	n.a.	n.a.	n.d.
*Togaviridae*	Liver	*neg.* (Chikungunya virus)	n.a.	n.a.	n.a.	n.d.
*Poxviridae*	Liver	*neg.* (Cowpox virus)	n.a.	n.a.	n.a.	n.d.
*Flaviviridae*	Liver	*neg.* (Rat hepacivirus)	n.a.	n.a.	n.a.	n.d.
*Hepeviridae*	Liver	*neg.* (Rat hepatitis E virus)	n.a.	n.a.	n.a.	n.d.
*Togaviridae*	Liver	*neg.* (Sindbis virus)	n.a.	n.a.	n.a.	n.d.
*Togaviridae*	Liver	*neg.* (Venezuelan equine encephalitis virus )	n.a.	n.a.	n.a.	n.d.
*Togaviridae*	Liver	*neg.* (Western equine encephalitis virus)	n.a.	n.a.	n.a.	n.d.
*Flaviviridae*	Liver	*neg.* (Zikavirus)	n.a.	n.a.	n.a.	n.d.
*Morganella*	Lung	n.a.	n.a.	n.a.	n.a.	*pos.* (*Morganella* sp.)
*Arenaviridae*	Lung	*neg.* (Arenavirus)	n.a.	n.a.	n.a.	n.d.
*Hantaviridae*	Lung	*neg.* (Seoul orthohantavirus)	n.a.	n.a.	n.a.	n.d.
*Staphylococcus*	Nose	*pos.* (*Staphylococcus aureus*)*	n.a.	n.a.	n.a.	n.a.
*Streptobacillus*	Pharyngeal swab	*neg.* (*Streptobacillus* sp.)	n.a.	n.a.	n.a.	n.a.
*Anaplasma*	Spleen	*neg.* (*Anaplasma phagocytophilum*)	n.a.	n.a.	n.a.	n.d.
*Bartonella*	Spleen	*neg.* (*Bartonella* sp.)	n.a.	n.a.	n.a.	n.d.
*Neoehrlichia*	Spleen	*neg.* (*Neoehrlicha mikurensis*)	n.a.	n.a.	n.a.	n.d.
*Arenaviridae*	Spleen	*neg.* (Arenavirus)	n.a.	n.a.	n.a.	n.d.
*Polyomaviridae*	Spleen	*neg.* (*Rattus norvegicus* Polyomavirus 1)	n.a.	n.a.	n.a.	n.d.
*Babesia*	Spleen	*neg.* (*Babesia* sp.)	n.a.	n.a.	n.a.	n.d.
*Streptobacillus*	Tongue	*neg.* (*Streptobacillus* sp.)	n.a.	n.a.	n.a.	n.a.

n.a., not analyzed.

n.d., not detected.

* PCR from cultivated colony.

*pos*., positive.

*neg*., negative.

For open-view analysis, a metagenomics approach was performed using HTS. The HTS produced a total number of reads between 1,642,880 to 9,145,736 per sample, with a proportion of 97.15% − 99.19% high quality (HQ) reads; of these HQ reads 98.4% − 99.99% were classified (see Table S2). The highest proportion of reads of the CCF and tissue libraries were classified as eukaryote-derived sequences (99.73% − 99.99%), except the feces-derived library with 44.45% (Table S3). As indicated in Table S3, the number of viral reads was typically low, except in the feces sample. In the feces sample, the number of bacterial reads was also much higher than in the tissue and CCF samples.

In parallel, two fecal sample-based isolation attempts, with subsequent MALDI-TOF MS analyses, resulted in the isolation and identification of eight bacterial species, i.e. two *Lactobacillus* spp., one *Ligilactobacillus* sp., *Enterobacter cloacae*, *Cellulosimicrobium cellulans*,* Brucella* (*Ochrobactrum*) *tritici*, *S. aureus*, *Klebsiella aerogenes*, and two fungal species, namely *Rhodotorula mucilaginosa* and *Cutaneotrichosporon mucoides* (Table 1). The *Lactobacillus* species were detected by both isolation approaches and were found in the feces HTS dataset (Table 1). The detection of *Ligilactobacillus* sp. by one isolation, and subsequent MALDI-TOF MS identification, was supported by HTS analysis of the fecal sample (Table 1). *Enterobacter* spp. and *Brucella* sp. were exclusively detected by the cultivation-MALDI-TOF MS approach, but not found by HTS (Table 1).

*S. aureus* was isolated from the rat nose, which is known to be the major niche for this microbial species, and the same species and strain (see below) was also isolated by culturing the fecal sample (Table 1).

Analysis of the HTS datasets revealed reads of additional bacteria, namely *Acinetobacter* sp. (CCF and feces), but *Acinetobacter* sp. was not detected by an isolation approach of the tracheal sample (Table 1). In addition, reads were assigned to *Streptococcus* sp., *Morganella* sp., *Clostridioides* sp., but without detection by any other approach (see Table 1).

Concerning fungi, isolation and MALDI-TOF MS-based detection of *Rhodotorula mucilaginosa* was accompanied by the detection of *Rhodotorula* sp. in the feces HTS data set (Table 1). MALDI-TOF MS-based identification of *Cutaneotrichosporon mucoides* was confirmed by 18S rRNA gene sequencing, but was not found in the HTS datasets (Table 1). The 18S rRNA gene sequence of the fungus *Cystobasidium* sp. was extracted from the HTS dataset (L2211; Table 1) and showed an identity of 99.8% to *Cystobasidium pinicola* and 99.8% to *Cystobasidium laryngis.*

### Characterization and WGS of *Staphylococcus aureus*

Both the nasal and fecal *S. aureus* isolates were methicillin-sensitive *S. aureus* (MSSA) and belonged to a novel *spa* type (t16921, clonal complex CC45). A subsequent DNA array-based characterization of the nasal isolate revealed superantigen genes, which are characteristic for CC45 isolates (enterotoxin gene cluster; *egc*), as well as the lineage-specific *agr* type 1. Moreover, the rat CC45-MSSA strain showed a high degree of similarity with human CC45-MSSA strains (Fig. 1B). The only discrepancies were the Mobile Genetic Elements (MGE)-encoded superantigens SEC and SEL, present in 67.9% of human isolates, but absent in the rat strain, and the adhesin bone sialoprotein-binding protein (BBP), present in 80.4% of the human strains but absent in the rat isolate. Notably, both rat strains harbored a hemolysin beta (*hlb*)-integrating phage encoding a human-specific immune evasion gene cluster (IEC) carrying staphylokinase (SAK), staphylococcal complement inhibitor (SCN) and chemotaxis inhibitory protein of *S. aureus* (CHIPS; CHP)^66,67^. As expected, this phage was highly prevalent in the human CC45-MSSA strain collection (96.4%). The only antibiotic resistance encoded by the rat isolate was β-lactamase conferring ampicillin resistance, which was also common among human isolates (39.3%).

**Fig. 3:**
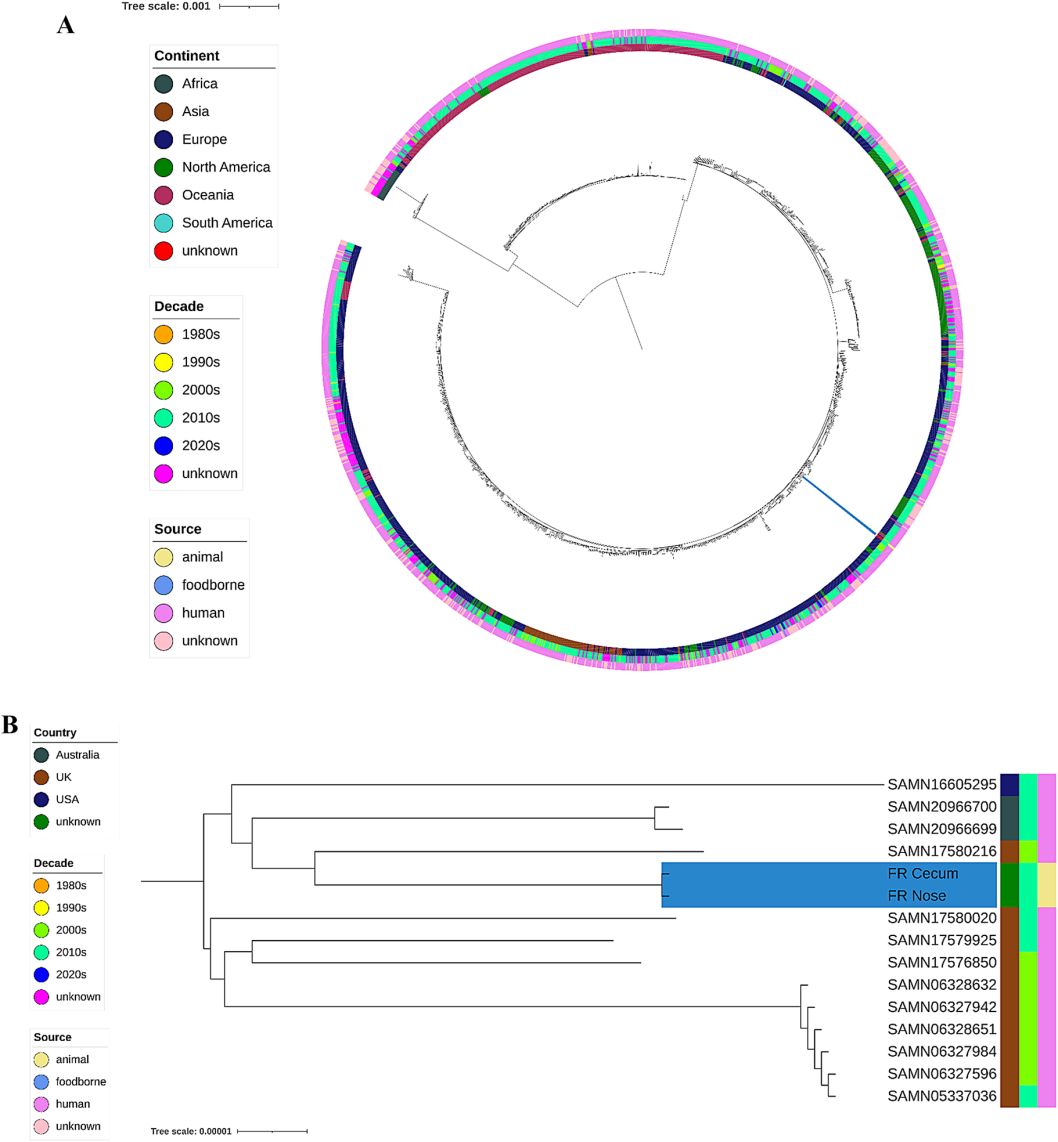
Phylogenetic relationship of the two MSSA isolates (blue, bottom right) within the context of published ST45 genomes (*n* = 1,689) visualized in iTOL (**A**). A core genome of 1,528 genes present in 99% of the collection was selected, and sample-wise alignments of the gene sequences were used to infer a phylogenetic tree using a maximum likelihood method. The resulting tree was midpoint rooted. For each sample, the metadata available through Pathogenwatch is illustrated as rings surrounding the phylogeny, containing (from inner to outer rings): geographic origin, decade and source. The MSSA isolates illustrated close genomic similarity to samples from the United Kingdom (UK), United States of America (USA) and Australia (**B**).

To assess the phylogenetic origin of the nasal and fecal isolates, WGS was performed. By combining both short- and long-read sequencing data, two closed genomes of the rat ST45 MSSA strains were fully reconstructed, with sizes of 2,746,620 bp (cecum) and 2,746,639 bp (nasal) for their chromosomal sequences, respectively. Both genomes further contained a Rep3-Inc18 family-like plasmid of 41,450 bp (cecum) and 41,451 bp (nasal) length. Comparison of these genomes at the nucleotide level revealed near-identical genetic composition (Figure S2), with only two minor ﻿SNP identified within the *pts*G gene (missense_variant c.73G > A p.Ala25Thr) and a non-coding region within the plasmidal sequences. A comparison of the rat MSSA with a collection of 1,692 publicly available ST45 samples provided additional insights into its phylogenetic origin (Fig. 3A). These strains represented a collection of both MSSA (*n* = 1114) and MRSA (*n* = 575) isolates based on in silico AMR profiling. The rat MSSA was closely related to isolates from human sources, primarily from Europe, but also illustrating genetic similarity to few samples from North America and Oceania. In particular, genomic similarities existed (ANI of 99.80 to 99.93) with a cluster of human-associated isolates from the United Kingdom (UK), United States of America (USA), and Australia, collected between 2008 and 2016 (Fig. 3B).

### Detection and characterization of PBVs

The RIEMS analysis of the fecal sample (L2211) assigned 182 reads to the family *Picobirnaviridae.* The sequence reads could be assembled to two segment 1 and two segment 2 sequences of PBV (Figure S3). Due to the co-detection of two segment 1 sequences and two segment 2 sequences in the rat feces sample, each segment was treated as a separate entity (PBV1-PBV4). The PBV segment 1 sequences had lengths of 2,773 bp (PBV1) and 2,528 bp (PBV3); the PBV segment 2 sequences were 1,752 bp (PBV2) and 1,630 bp (PBV4) (see Figure S3C, Table S4). The genome organization was similar to a prototype member of this virus family, as well as two Norway rat-associated PBV strains from Berlin (Table S4; Figure S3A and S3B). The open reading frame (ORF) on the segment 2 encodes a RdRp of 451–532 amino acid residues, and the ORFs on the segment 1 encode two putative proteins of 206–237 and 546–631 amino acid residues (Table S4). The four sequences had varying coverages, namely PBV1 median coverage 16 (inter quartile range (IQR)12), PBV2 median coverage 28 (IQR 19), PBV3 median coverage 4 (IQR 4), PBV4 median coverage 6 (IQR 10).

For comparison, PBV sequences were assembled from the two Norway rats from Berlin^18^, showing segment 1 sequences of 2,674 and 2,523 bp and segment 2 sequences of 1,385 and 1,656 bp (Figure S3B). The segment 1 sequences indicated two ORFs, whereas the segment 2 sequences showed only a single ORF, encoding the RdRp. For the two Berlin rats, and one of the stowaway rat-derived PBV sequences, the two ORFs on the segment 1 were partially overlapping (Figure S3B, and C).

**Fig. 4:**
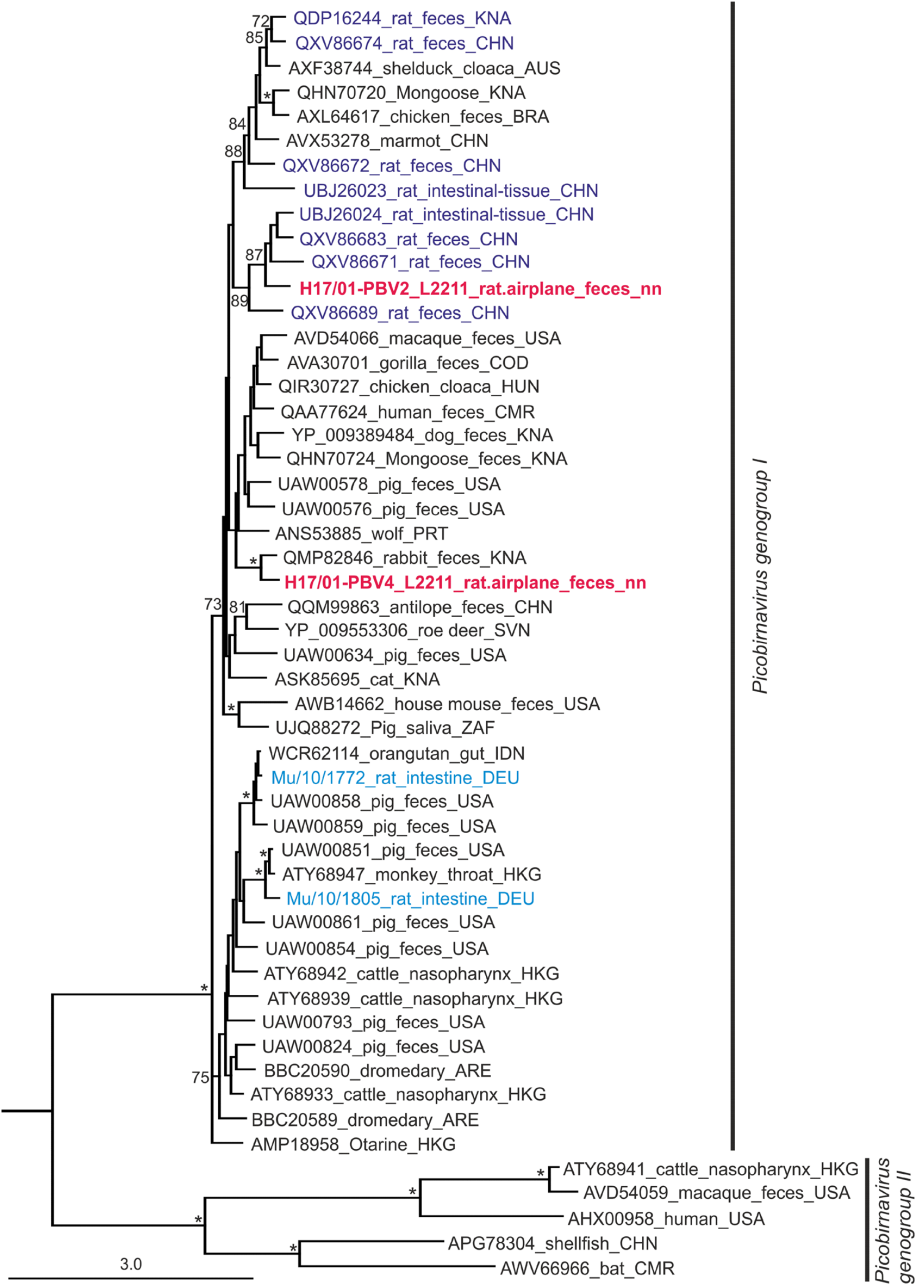
Phylogenetic relationships of picobirnavirus (PBV) strains detected in this study using RNA-dependent RNA polymerase amino acid sequences. The tree was constructed using the maximum likelihood method. Values for 500 bootstrap replications above 70% are shown, with asterisks for values above 90%. Rat-derived sequences are given in blue, including PBV sequences from Norway rats in Berlin (DEU) in light blue; novel PBV sequences from the airplane rat are given in red. Beihai picobirna-like virus 6 (APG78167) was used as outgroup taxon (not shown). Country codes (ISO 3166-1 alpha-3): ARE, United Arab Emirates; AUS, Australia; BRA, Brazil; CHN, China; CMR, Cameroon; COD, Congo; DEU, Germany; HKG, Hong Kong; HUN, Hungary; IDN, Indonesia; KNA, Saint Kitts and Nevis; PRT, Portugal; SVN, Slovenia; USA, United States of America; ZAF, South Africa; nn, not known.

For phylogenetic analysis, we used exclusively the RdRp amino acid sequences, as the sequence variability for the other proteins was too high. Based on the RdRp amino acid sequence, the phylogenetic tree showed that both airplane rat-derived RdRp sequences (H17/01-PBV2 and -PBV4) clustered within PBV genogroup I (Fig. 4). One of the sequences (H17/01-PBV2) clustered within a group consisting of sequences from rat fecal and intestine samples from China, whereas the other (H17/01-PBV4) was related to samples from other animals (closest related sample comes from a rabbit). The sequences of Norway rats trapped in Berlin (Mu/10/1805 and Mu/10/1772)^18^, additionally analyzed in this study, did not show a close relationship to the H17/01-airplane PBV sequences, but grouped together with pig feces samples from a pig farm in the USA in another subgroup (Fig. 4).

### Pathogen-specific methods and multiplex serology

Pathogen-specific RT-PCR and PCR did not detect any of the tested 20 viral pathogens, ten bacterial pathogens and one endoparasite (Table 1).

The Luminex-based multiplex serology targeted four bacterial species and 13 viruses, and also included additional related viruses through the use of cross-reactive antigens of rat parvoviruses (NS1 antigen) and rodent orthohantaviruses (nucleocapsid protein). The mean MFI values ranged from 1 to 28, indicating a lack of pathogen-specific serum IgM and/or IgG antibodies (Table 1).

Where pathogen-specific methods (PCR, RT-PCR and serology) indicated the absence of a virus or bacterium, this was further supported by the absence of pathogen-specific reads in reference-mapped HTS datasets. However, HTS datasets did contain reads for *Acinetobacter* sp. in CCF and feces, and *Clostridioides* sp. in feces, which were not detected using pathogen-specific methods (Table 1).

## Discussion

The global transport of animals (intentional or unintentional) can facilitate the incursion of pathogens into novel regions^68^. There are various examples of the associated intercontinental incursion of rodent-borne pathogens, e.g. squirrel poxvirus with the grey squirrel (*Sciurus carolinensis*)^69^. The introduction of the grey squirrel from Northern America to Great Britain, Ireland and Italy caused a massive decline in the indigenous red squirrel populations, most likely caused by the incursion of squirrel poxvirus^70^. Other rodent species were introduced intentionally into Europe, e.g. muskrats (*Ondatra zibethicus*) and coypu (*Myocastor coypus*) for fur production. These invasive species also represent reservoirs for zoonotic pathogens^71^.

According to IATA and WHO regulations, rodents on board of an airplane must be eliminated immediately^72^. Extensive checks, inspections, and hygiene measures must be carried out, and rodent damage must be repaired, if necessary. Within the scope of pest infestation analysis, the exclusion of dangerous infectious pathogens, as well as a proof of the effectiveness of a CO_2_ fumigation, must be provided. In accordance with air traffic regulations, if the comprehensive pathogen screening described here, particularly for highly pathogenic air-borne viruses such as Sin nombre orthohantavirus^73^, had detected a pathogen of interest, a trace-forward screening of passengers, and the fumigation and disinfection of the airplane would have been required.

To evaluate the potential origin of the black rat, i.e. the United Arab Emirates (Dubai) or USA, Florida (Miami), the complete cytochrome *b* and mtDNA sequences were determined and compared to published sequence data from black rats of different origins. The rat cytochrome *b* sequence was classified as black rat lineage 1, which is distributed worldwide. Due to the lack of phylogeographic structure within the lineage 1 mtDNA sequences for black rats from various regions, including the potential geographic origins of the rat in question, it was not possible to resolve the origin of the rat.

We further screened the black rat for potentially zoonotic pathogens or other infectious agents using untargeted open-view, multiplex and pathogen-specific methods. Taken together, the open-view methods detected a few infectious agents, some of which were potentially zoonotic, and numerous, often commensal bacterial species, whereas multiplex serology and all pathogen-specific approaches did not detect any infectious agent. The combination of bacterial culture with MALDI-TOF MS, as a rapid procedure, and the parallel metagenomics approach facilitated the identification of several bacteria and fungi. Although the results obtained by the two classical microbiology-MALDI TOF MS analyses were not completely identical, there was significant agreement, e.g. detection of *Lactobacillus* spp. and *Enterobacter* spp. in each approach. In addition, HTS analyses of feces samples confirmed the cultivation and MALDI-TOF MS-based detection of *Lactobacillus* sp. and *Ligilactobacillus* sp. The observed discrepancies between the two cultivation-MALDI-TOF MS approaches and between cultivation-MALDI-TOF MS and the HTS approach can be attributed to the differences in the sensitivity of the methods, and to varying distribution of bacteria in the individual intestinal sections, i.e. the inhomogeneous distribution in feces, but also in the subculture that precedes the species identification, and the quality of the starting material for cultivation, i.e. consequences of freeze-thaw cycles. To circumvent the cultivation-related problems, the HTS-based pathogen detection might be used in the future for highly sensitive detection of known, but also so far unknown pathogens. Nevertheless, in future investigations, the storage of samples for bacteria and fungi isolation approaches should be optimized to prevent freeze-thaw-cycles by using transport media.

Taken together, the cultivation-MALDI-TOF MS and HTS investigations detected mainly commensal bacteria, i.e. *Lactobacillus* spp., *Ligilactobacillus* sp., *Morganella* sp., *Clostridioides* sp., *Streptococcus* sp., *Acinetobacter* sp., *Enterobacter* spp., *Brucella* (*Ochrobactrum*) sp. and *Cellulosimicrobium cellulans* and commensal fungi, i.e. *Rhodotorula mucilaginosa*. Lactobacilli are typical commensal members of the mammal/rat intestine^74^. Therefore, the *Lactobacillus* spp. and *Ligilactobacillus* sp. found in the feces of the rat are most likely commensals of the rat microbiota. Similarly, *Enterobacter* species are part of the intestinal microbiota of humans and animals, but can also be etiologically relevant in human infections, for example as nosocomial pathogens carrying multiple antimicrobial resistance genes^75^. Streptococci are a large group of Gram-positive bacteria including pathogenic and non-pathogenic species of various mammalian hosts. Typically, they are part of the oral and upper respiratory tract microbiome. *Cellulosimicrobium cellulans* is a Gram-positive bacterium found in soil and wastewater. It can rarely cause opportunistic infections in immunocompromised patients^76^. *Rhodotorula mucilaginosa* is a yeast commonly found in soil and wastewater. It can also colonize human mucosa as a commensal organism. On rare occasions, it causes fungemia in immunosuppressed individuals^77^. The detection of some of these commensal agents at unexpected locations of the rat, e.g. streptococci reads in CCF, might be caused by cross-contamination during dissection.

We also detected a methicillin-susceptible *S. aureus* strain of the MLST clonal complex 45 (MSSA-CC45) in both nose and feces samples. Such CC45-MSSA strains are highly prevalent in the human population^34^. We have previously reported that mouse-adapted *S. aureus* isolates differ from human strains in three aspects: (I) they lack MGE-encoded superantigen genes, (II) they lack the *hlb*-integrating phage encoding a human specific immune evasion cluster, and (III) they lack ampicillin resistance^28,78^. The nasal *S. aureus* isolate from the airplane rat lacked the MGE-encoded superantigens SEC and SEL, but was ampicillin-resistant. Moreover, it harbored the *hlb*-phage-encoded human-specific immune evasion cluster, which likely confers adaptation to the human host^79^. Collectively, these data suggest a recent transmission from humans to this rat.

In order to assess the origin of these samples, WGS was performed on both the nasal and fecal *S. aureus* isolate. Two complete genomes of high quality were reconstructed by combining the accuracy of short-read sequencing with long-read ONT sequencing. WGS hereby revealed an overall high genetic identity (SNP distance of 2) between both genomes, as well as the presence of a Rep3-Inc18 family-like plasmid. Further phylogenetic analyses revealed close genetic relationships with strains originating from multiple sources, including a closely related cluster of human-associated isolates collected from the UK between 2008 and 2016. However, attribution of a country of origin of the MSSA strain based on the WGS data remained particularly challenging, as public data sources are often limited in their accompanying metadata and databases may suffer from submission biases. Nevertheless, other studies demonstrate that rats can play an important role in the transmission and spread of *S. aureus* to humans^80,81^. Rats living in close vicinity to humans often carry human MRSA lineages, while rats without contact to humans and farm animals are colonized with *S. aureus* lineages that also occur in other wild animals^22,80,81^. This suggests that rats are an important reservoir of *S. aureus* and in particular MRSA for humans and livestock.

Concerning viruses, we detected novel PBV sequences in the feces of the black rat by HTS. The simultaneous detection of fungi within the feces of the black rat might be related to the association of PBVs with fungi as recently discussed^82^. Due to the co-detection of two segments 1 and two segments 2 within the feces sample of the rat, we were unable to define their association within a virus particle and treated all of them as separate entities (PBV1-PBV4). The almost complete genome segments showed the typical PBV genome structure, i.e. two ORFs on segment 1 and a single RdRp-encoding ORF on segment 2. We found no evidence for non-segmented PBV as has previously been reported in mammals and birds^83–85^. Despite the general similarity of the PBV genome organization of the airplane rat and the Norway rats collected in Berlin, two obvious differences exist: (I) in three of four dsRNA1 segments the two ORFs do not overlap, but in one they do, (II) the length of the genome segments and of the ORFs differ. The amino acid sequence of the RdRp illustrates the high level of sequence variability even within rat-associated PBV strains. Moreover, the phylogenetic investigation of the amino acid sequences of the RdRp showed a very different position of the rat feces-derived strains within the phylogenetic tree, with similarity of some of them (Berlin rats) to strains found in pig feces^86^.

The comprehensive investigation of the rat revealed a low risk for human health as the majority of detected infectious agents were commensals. Neither rat-associated zoonotic pathogens, i.e. *Leptospira* spp., *Streptobacillus* spp., SEOV or ratHEV nor additional rat-specific pathogens were identified by multiplex serology, pathogen-specific assays, or HTS.

## Conclusions

We, here, developed a comprehensive workflow to investigate whether or not this animal was carrying pathogens which could potentially harm the passengers. The combination of a broad spectrum of methods, untargeted open-view, pathogen-specific and multiplex methods combined with WGS allows a comprehensive identification of potential pathogens within an incursion scenario and a data-driven risk assessment. The detection of an MSSA closely related to strains of human origin stresses the need for improved surveillance as well as containment measures. The detection of different PBV strains in the black rat, but also in rats of another origin, suggests a broad distribution of these viruses. Future investigations should evaluate the reservoir(s) of PBV, as our study could not verify the rat as a reservoir host. In particular, the potential co-occurrence of PBVs and specific fungi or bacterial species needs to be proven for its role in virus transmission.

## Supplementary Information

Below is the link to the electronic supplementary material.

Supplementary Material 1

## Data Availability

Data is provided within the manuscript or supplementary information files. Sequence data that support the findings of this study have been deposited with the Study Accession PRJEB82892; WGS of the two MSSA strains were uploaded at NCBI with Bioproject ID: PRJNA1118235. To request data from our study, Claudia Wylezich and Dirk Höper (Study Accession PRJEB82892) and Silver A. Wolf and Torsten Semmler (Bioproject ID: PRJNA1118235) or the corresponding author should be contacted.
