# Supplementary material for: Application of a comprehensive approach to pathogen screening in a stowaway rat on an airplane
Source: Sci Rep. 2025 Aug 30;15:31963. doi: 10.1038/s41598-025-13199-6 (PMC12398574; doi:10.1038/s41598-025-13199-6)
Supplement: Supplementary file 1 — Supplementary Material 1 [file 41598_2025_13199_MOESM1_ESM.docx]

**Supplementary Material**

**Application of a comprehensive approach to pathogen screening in a stowaway rat on an airplane**

Elisa Heuser^1^, Arnt Ebinger^2^, Silva Holtfreter^3^, Silver A. Wolf^4^, Andreas E. Zautner^5,18^, René Ryll^1^, Stephan Drewes^1^, Beate Matzkeit^1^, Bernd Hoffmann^2^, Dirk Höper^2^, Markus Keller^1^, Allison Groseth^6^, Gottfried Wilharm^7^, Daniel M. Mrochen^3^, Anna Obiegala^8^, Frank Doss^9^, Calvin Mehl^1,17^, Tobias Eisenberg^10^, Sandra Niendorf^11^, Sindy Böttcher^12^, Axel Karger^6^, Charlotte Schröder^13^, Eric Ehrke-Schulz^14^, Katja Schmidt^15^, Martin Beer^2^, Martin H. Groschup^1^, Torsten Semmler^4^, Gerald Heckel^16^, Martin Pfeffer^8^, Claudia Wylezich^2,13^ & Rainer G. Ulrich^1,17,*^

^1^ Institute of Novel and Emerging Infectious Diseases, Friedrich-Loeffler-Institut, Federal Research Institute for Animal Health, Südufer 10, 17493 Greifswald-Insel Riems, Germany

^2^ Institute of Diagnostic Virology, Friedrich-Loeffler-Institut, Federal Research Institute for Animal Health, Südufer 10, 17493 Greifswald-Insel Riems, Germany

^3^ Institute of Immunology, University Medicine Greifswald, 17475 Greifswald, Germany

^4^ Robert Koch Institute, Genome Competence Centre (MF1), Nordufer 20, 13353 Berlin, Germany

^5^ Institut für Medizinische Mikrobiologie und Virologie, Universitätsmedizin Göttingen (UG), Kreuzbergring 57, 37075 Göttingen, Germany

^6^ Institute of Molecular Virology and Cell Biology, Friedrich-Loeffler-Institut, Federal Research Institute for Animal Health, Südufer 10, 17493 Greifswald-Insel Riems, Germany

^7^ Robert Koch Institute, Wernigerode Branch, Burgstr. 37, D-38855 Wernigerode, Germany

^8^ Institute of Animal Hygiene and Veterinary Public Health, University of Leipzig, An den Tierkliniken 1, 04103 Leipzig, Germany

^9^ clean Frank Doss GmbH, Kampstraße 27, 16792 Zehdenick, Germany

^10^ Landesbetrieb Hessisches Landeslabor (LHL), Schubertstraße 60 - Haus 13, 35392 Gießen, Germany

^11^ Robert Koch Institute, Department of Viral Gastroenteritis and Hepatitis Pathogens and Enteroviruses, Seestraße 10, 13353 Berlin, Germany

^12^ Robert Koch Institute, Regional Reference Laboratory of the WHO/Europe for Poliomyelitis, Seestraße 10, 13353 Berlin, Germany

^13^ Department of Experimental Animal Facilities and Biorisk Management, Friedrich-Loeffler-Institut, Federal Research Institute for Animal Health, Südufer 10, 17493 Greifswald-Insel Riems, Germany

^14^ Lehrstuhl für Virologie und Mikrobiologie, Zentrum für biomedizinische Ausbildung und Forschung (ZBAF), Universität Witten/Herdecke, Stockumer Strasse 10, 58453 Witten, Germany

^15^ German Cancer Research Center Heidelberg, Microbiological Diagnostics, Im Neuenheimer Feld 280, 69120 Heidelberg, Germany

^16^ Institute of Ecology and Evolution, University of Bern, Baltzerstrasse 6, 3012 Bern, Switzerland

^17^ German Centre for Infection Research (DZIF), Partner Site Hamburg-Lübeck-Borstel-Riems, Greifswald-Insel Riems, Germany

^18^ Current affiliation: Universitätsklinikum Magdeburg A. ö. R., Institut für Medizinische Mikrobiologie und Krankenhaushygiene, Leipziger Str. 44, 39120 Magdeburg

*Corresponding author.


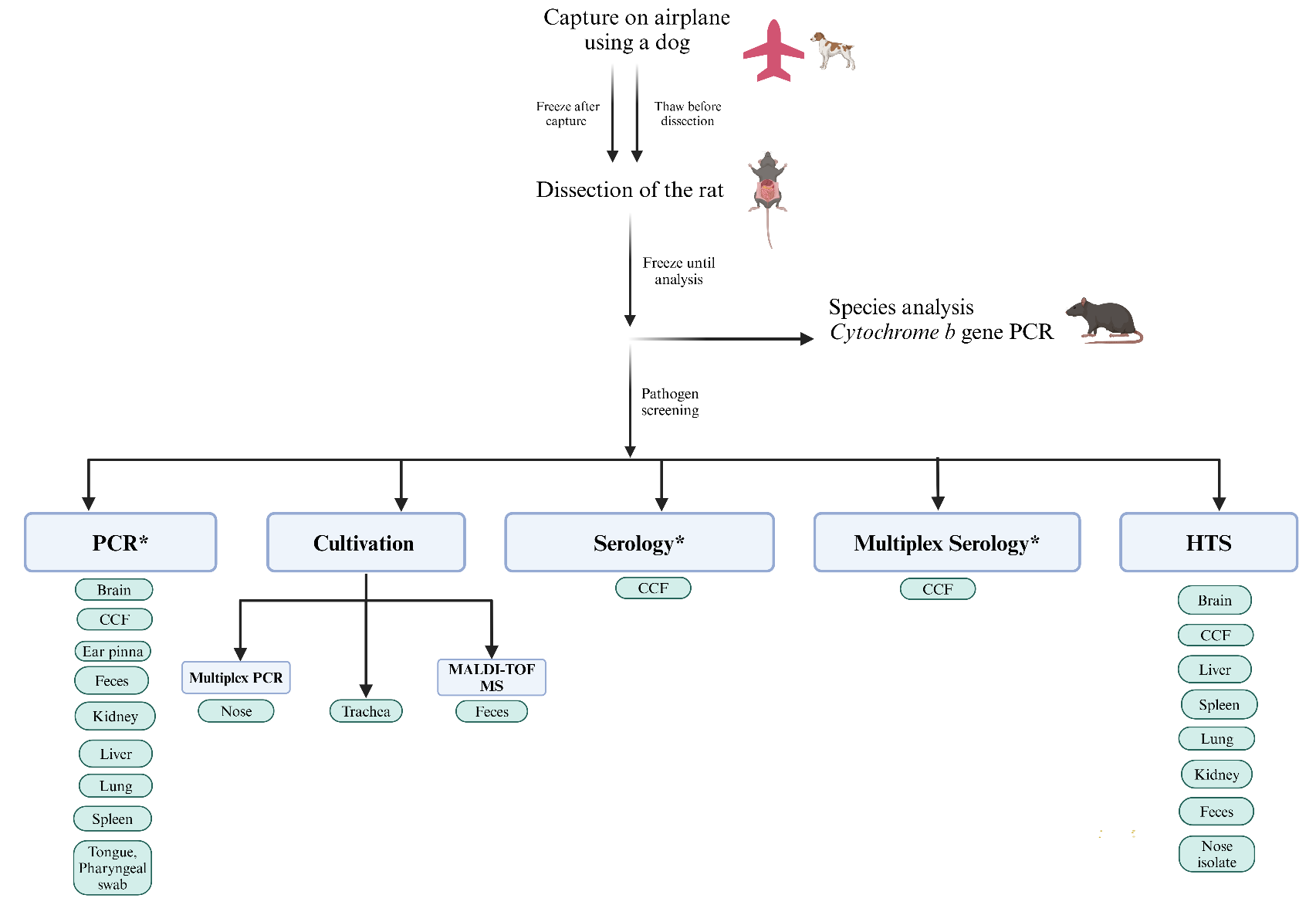


**Fig. S1.** Workflow of the collection, dissection and investigations of the rat (H17/01) detected in an airplane; created with Biorender (license Elisa Heuser). Legend: CCF, chest cavity fluid; HTS, high-throughput sequencing; MALDI-TOF MS, Matrix assisted laser desorption ionization-Time of flight mass spectrometry; PCR, polymerase-chain reaction; *for assay specificity see Table 1 and Table S1.


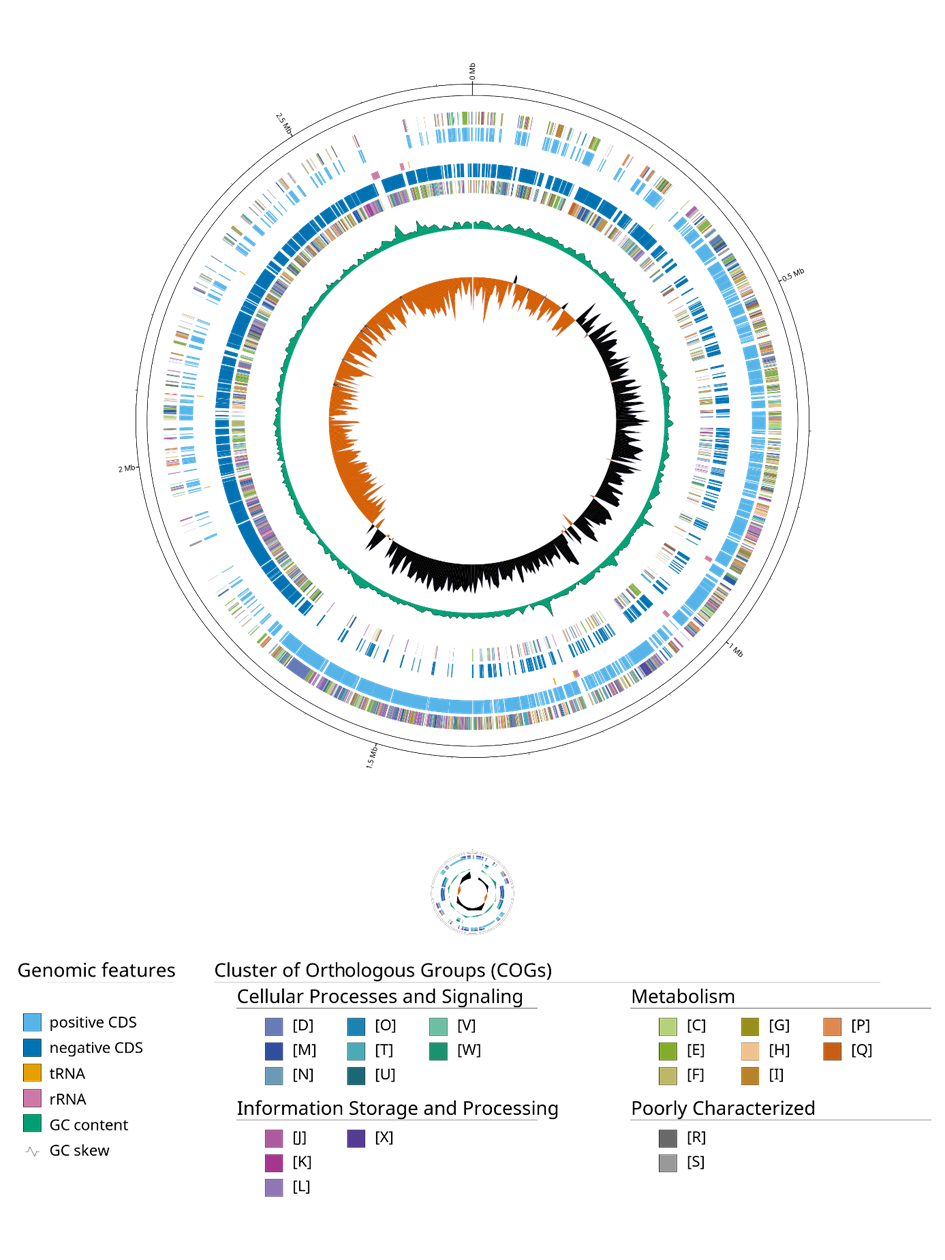


**Fig. S2.** Circular genomic representation of the MSSA genome created in GenoVi. Illustrated is only the nasal MSSA isolate, as the genome of the cecum MSSA was near-identical in size and structure (data not shown). Through a combination of long- and short-read sequencing technologies, both the chromosome (top) and plasmid sequence (bottom) of the strains could be fully reconstructed. The coloured rings represent (from outer to inner ring): contig, genomic features, strandedness, annotated COG terms, GC content and GC skew.

| **Segment 1** | **Segment 2** |
| --- | --- |
| **A) Human picobirnavirus**  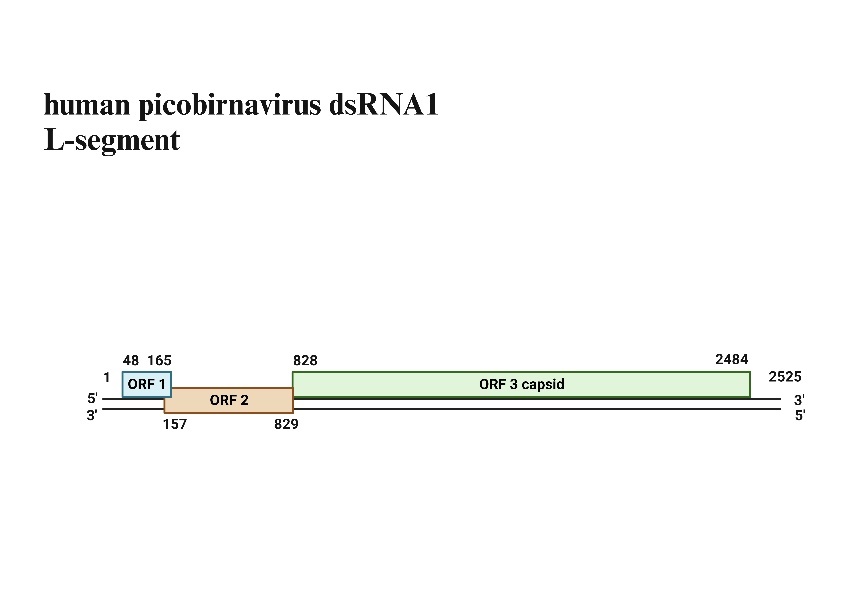 | 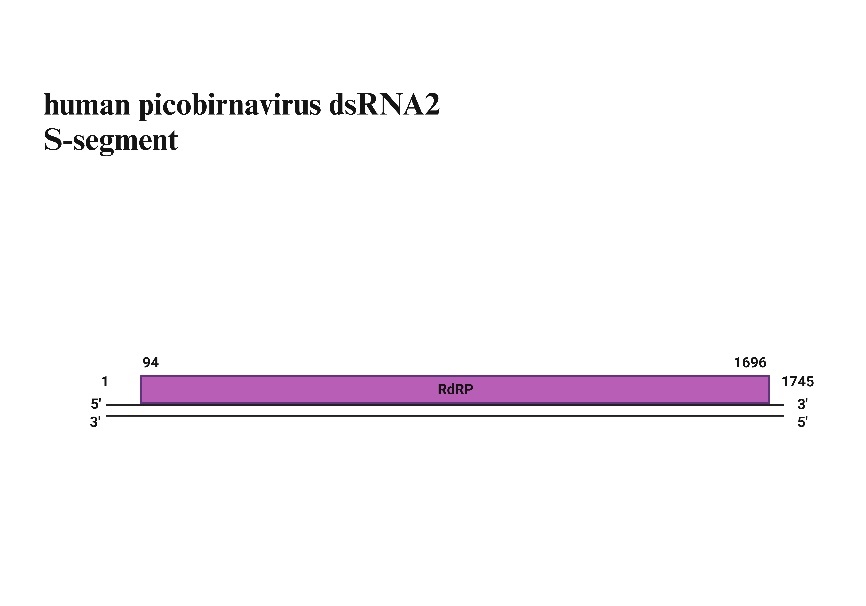 |
| **B) Rat Mu/10/1772 picobirnavirus**  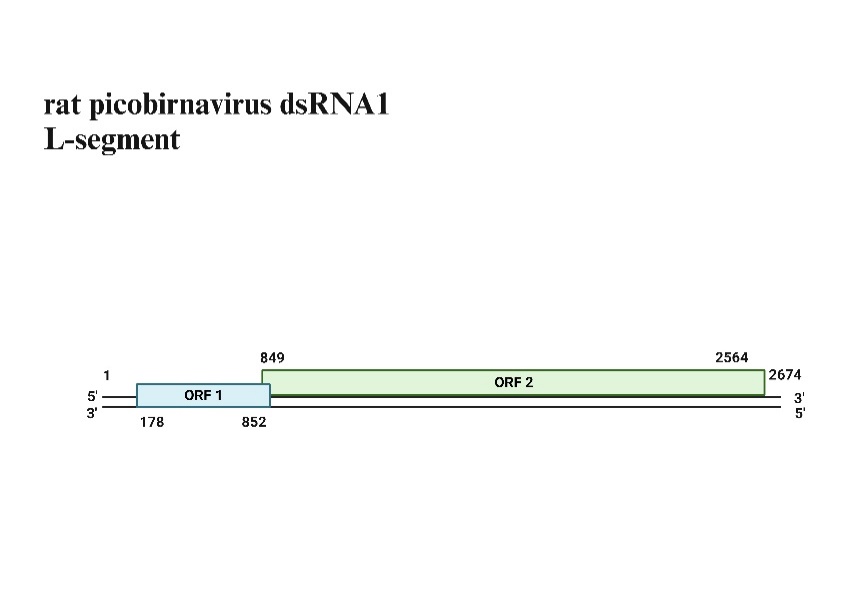 | 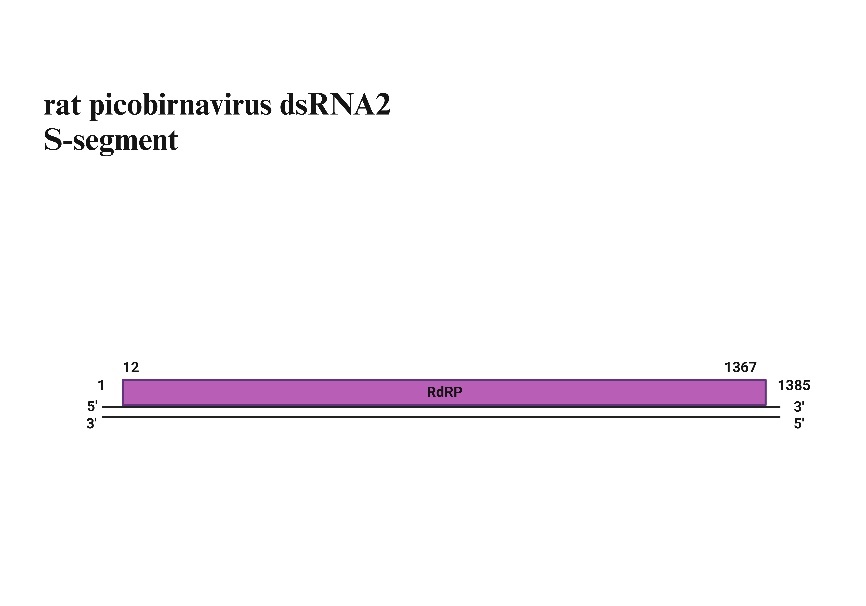 |
| 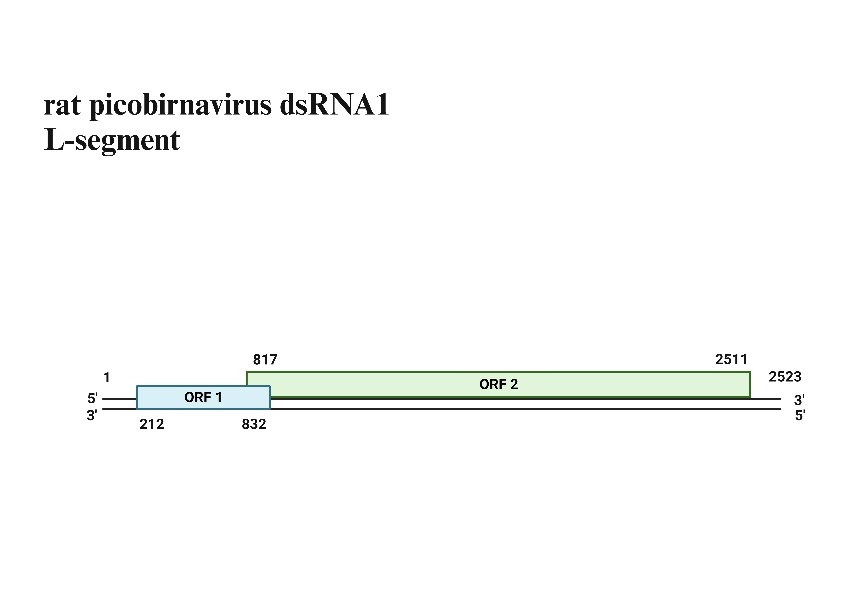 **Rat Mu/10/1805 picobirnavirus** | 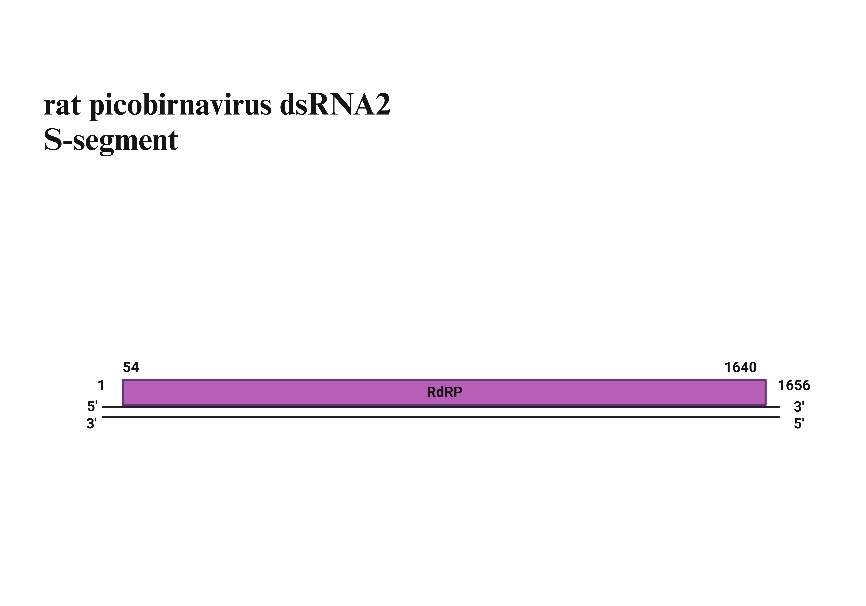 |
| **C) Rat H17/01 airplane picobirnavirus 1**  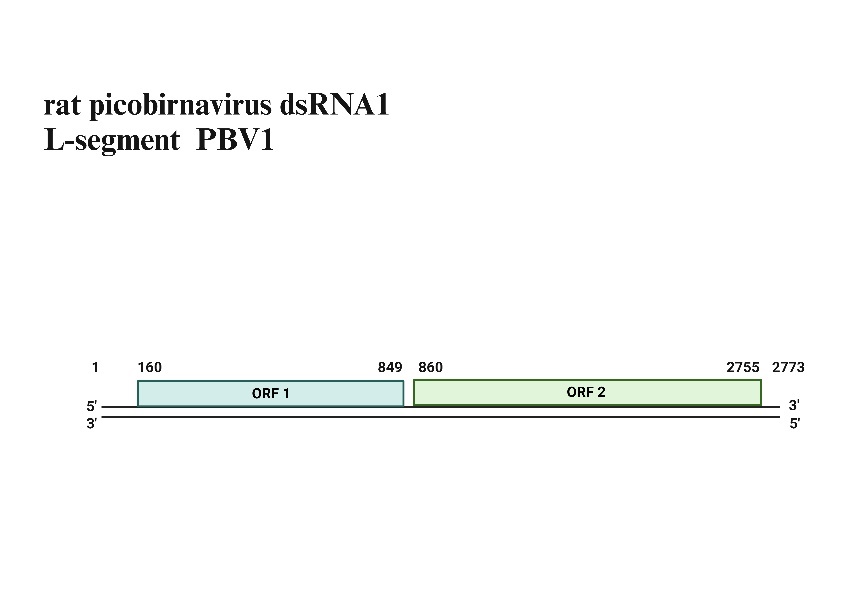 | **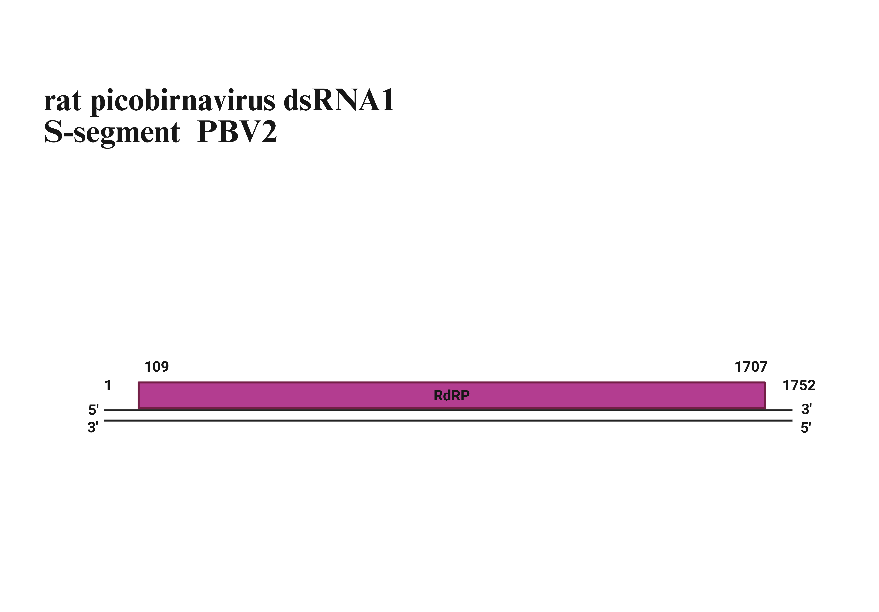Rat H17/01 airplane picobirnavirus 2** |
| **Rat H17/01 airplane picobirnavirus 3**  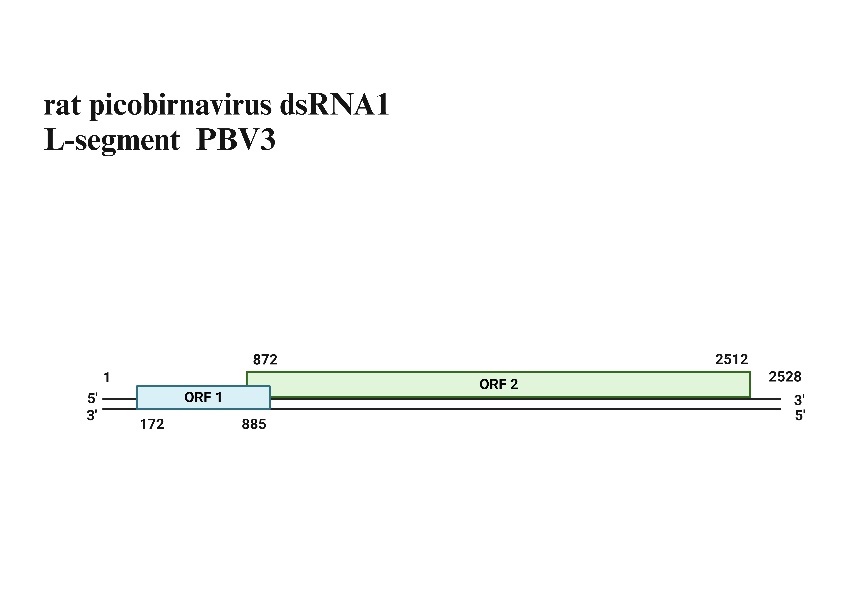 | **Rat H17/01 airplane picobirnavirus 4**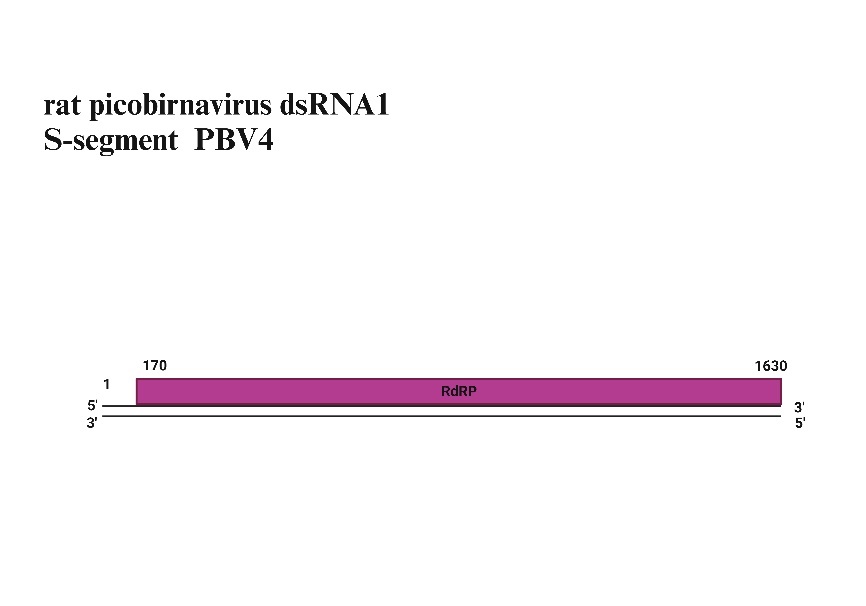 |

**Fig. S3.** Genome maps of prototype picobirnavirus (PBV) human strain HY005102 (segment 1: GenBank acc. no. NC 007026; segment 2: GenBank acc. no. NC 007027) (A), PBV strains from urban brown rats from Berlin Mu/10/1772 and Mu10/1805 (B) and genome maps of the HTS datasets for genome segments of rat H17/01-derived strains from the airplane (C). Of note, the association of the genome segments 1 and 2 to a virus could not be determined, therefore they were treated as independent entities. Genome maps created with Biorender (license Elisa Heuser).

**Table S1.** Methods from open view, multiplex and pathogen-specific methods.

|  | **Method** | | **Pathogen** | **References** |
| --- | --- | --- | --- | --- |
| **Open view** | Cultivation |  | *Acinetobacter* sp. | [1] |
|  |  | Multiplex PCR + HTS | *Staphylococcus* sp. | [2] |
|  |  | MALDI-TOF MS | Various | [3–7] |
|  | HTS | - | Various | [8] |
| **Multiplex systems** | Multiplex serology | - | Various | [9,10] |
| **Pathogen-specific nucleic acid detection** | PCR/RT-PCR based assays |  | *Leptospira* sp. | [11] |
|  |  |  | *Rattus norvegicus* Polyomavirus 1 | [12] |
|  |  |  | *Neoehrlichia* sp. | [13,14] |
|  |  |  | *Anaplasma* sp. | [14,15] |
|  |  |  | *Bartonella* sp. | [14] |
|  |  |  | *Babesia* sp. | [16] |
|  |  |  | Hantavirus | [17,18] |
|  |  |  | Rat hepacivirus | [19] |
|  |  |  | Cowpox virus | [20] |
|  |  |  | Rat hepatitis E virus | [21] |
|  |  |  | Papillomavirus | [22] |
|  |  |  | *Borrelia* sp. | [23] |
|  |  |  | *Rickettsia* sp. | [24] |
|  |  |  | Astrovirus | [25] |
|  |  |  | Bocavirus | [26] |
|  |  |  | Enterovirus | [27] |
|  |  |  | Sapovirus | [25] |

|  |  | **Method** | **Pathogen** | **References** |
| --- | --- | --- | --- | --- |
| **Pathogen-specific nucleic acid detection** | PCR/RT-PCR based assays |  | Rotavirus | [28] |
|  |  |  | Norovirus | [29] |
|  |  |  | Adenovirus | [30] |
|  |  |  | *Clostridioides* sp. | [31] |
|  |  |  | *Streptobacillus* sp. | [32] |
|  |  |  | Zika virus | [33] |
|  |  |  | Sindbis virus | [34] |
|  |  |  | Chikungunya virus | [35] |
|  |  |  | Eastern, Western, Venezuelan equine encephalitis virus | [36,37] |
|  |  |  | Arenavirus | [38,39] |
| **Pathogen specific serology** | ELISA |  | Eastern, Western, Venezuelan equine encephalitis virus | [40] |
|  |  |  | Rat hepatitis E virus | [41] |
|  |  |  | Hantavirus | [42] |

ELISA, enzyme-linked immunosorbent assay; HTS, high-throughput sequencing; MALDI-TOF MS, Matrix assisted laser desorption ionization-Time of flight mass spectrometry; PCR, polymerase chain reaction; RT-PCR, reverse transcription-PCR

**Table S2.** Overview of read numbers for datasets obtained for the RNA samples analysed by metagenomic next-generation sequencing (mNGS).

| **Sample ID** | **Library ID** | **Total Reads** | **HQ Reads** | **Classified Reads** | **Unclassified Reads** |
| --- | --- | --- | --- | --- | --- |
| H17/01 Liver | L2208 | 1,867,257 (100 %) | 1,815,520 (97.23 %) | 1,814,493 (99.94 %) | 1,027 (0.06 %) |
| H17/01 Spleen | L2209 | 1,706,774 (100 %) | 1,674,776 (98.13 %) | 1,674,545 (99.99 %) | 231 (0.01 %) |
| H17/01 Lung | L2210 | 3,856,330 (100 %) | 3,825,103 (99.19 %) | 3,824,643 (99.99 %) | 460 (0.01 %) |
| H17/01 Feces | L2211 | 9,145,736 (100 %) | 8,981,113 (98.2 %) | 8,837,079 (98.4 %) | 144,034 (1.6 %) |
| H17/01 CCF | L2212 | 1,642,880 (100 %) | 1,610,831 (98.05 %) | 1,610,614 (99.99 %) | 217 (0.01 %) |
| H17/01 Brain | L2213 | 1,804,586 (100 %) | 1,753,199 (97.15 %) | 1,748,147 (99.71 %) | 5,052 (0.29 %) |
| H17/01 Kidney | L2214 | 5,631,122 (100 %) | 5,589,012 (99.25 %) | 5,588,509 (99.99 %) | 503 (0.01 %) |

CCF, chest cavity fluid; HQ Reads, number of reads after quality filtering; Classified Reads, number of reads classified taxonomically by RIEMS-analysis; Unclassified Reads, number of reads without significant sequence identity with sequences present in the used database.

**Table S3.** Division of the classified reads (given in Table S2) into the kingdoms eukaryotes, bacteria, archaea, and viruses. Figures in brackets are the corresponding percentages of the number of classified reads.

| **Sample ID** | **Library ID** | **Eukaryotes** | **Bacteria** | **Archaea** | **Viruses** |
| --- | --- | --- | --- | --- | --- |
| H17/01 Liver | L2208 | 1,809,718 (99.74 %) | 4,772 (0.26 %) | 0 (0 %) | 3 (0 %) |
| H17/01 Spleen | L2209 | 1,671,759 (99.83 %) | 2,780 (0.17 %) | 0 (0 %) | 6 (0 %) |
| H17/01 Lung | L2210 | 3,822,746 (99.95 %) | 1,890 (0.05 %) | 1 (0 %) | 6 (0 %) |
| H17/01 Feces | L2211 | 3,927,944 (44.45 %) | 4,908,092 (55.54 %) | 285 (0 %) | 758 (0.01 %) |
| H17/01 CCF | L2212 | 1,606,311 (99.73 %) | 4,285 (0.27 %) | 0 (0 %) | 18 (0 %) |
| H17/01 Brain | L2213 | 1,747,855 (99.98 %) | 279 (0.02 %) | 0 (0 %) | 13 (0 %) |
| H17/01 Kidney | L2214 | 5,588,110 (99.99 %) | 270 (0 %) | 0 (0 %) | 129 (0 %) |

**Table S4.** Comparison of picobirnavirus (PBV) segments, open reading frame (ORF) and protein lengths of the rat from the airplane (H17/01) and other different animal species of PBV genogroups 1 to 3.

| **Sample ID** | **Strain** | **Host species** | **Scientific host name** | 1. **Genogroup of PBV** |  | | 1. **segment 1** | | | | | | 1. **segment 2** | |
| --- | --- | --- | --- | --- | --- | --- | --- | --- | --- | --- | --- | --- | --- | --- |
|  |  |  |  |  |  |  | 1. **ORF 1** | | 1. **ORF 2** | | 1. **ORF 3 (capsid)** | | 1. **RdRP** | |
|  |  |  |  |  | 1. **Origin** | | 1. **nt*** | 1. **aa** | 1. **nt*** | 1. **aa** | 1. **nt*** | 1. **aa** | 1. **nt*** | 1. **aa** |
| 1. H17/01 | 1. PBV 1 | 1. rat | 1. *Rattus rattus* | 1. 1 | 1. ? | | 1. - | 1. - | 1. 687 | 1. 229 | 1. 1893 | 1. 631 | 1. - | 1. - |
|  | 1. PBV 2 | 1. rat | 1. *Rattus rattus* | 1. 1 | 1. ? | | 1. - | 1. - | 1. - | 1. - | 1. - | 1. - | 1. 1596 | 1. 532 |
|  | 1. PBV 3 | 1. rat | 1. *Rattus rattus* | 1. 1 | 1. ? | | 1. - | 1. - | 1. 711 | 1. 237 | 1. 1638 | 1. 546 | 1. - | 1. - |
|  | PBV 4 | 1. rat | 1. *Rattus rattus* | 1. 1 | 1. ? | | 1. - | 1. - | 1. - | 1. - | 1. - | 1. - | 1. 1458 | 1. 486 |
| 1. Mu10/1772 | 1. PBV | 1. rat | 1. *Rattus norvegicus* | 1. 1 | 1. DEU | | 1. - | 1. - | 1. 672 | 1. 224 | 1. 1713 | 1. 571 | 1. 1353 | 1. 451 |
| 1. Mu10/1805 | 1. PBV | 1. rat | 1. *Rattus norvegicus* | 1. 1 | 1. DEU | | 1. - | 1. - | 1. 618 | 1. 206 | 1. 1692 | 1. 564 | 1. 1584 | 1. 528 |
| 1. AB186897; AB186898 | 1. Hy005102 | 1. human | 1. *Homo sapiens* | 1. 1 | 1. THA | | 1. 117 | 1. 39 | 1. 672 | 1. 224 | 1. 1656 | 1. 552 | 1. 1602 | 1. 534 |
| 1. LC110352; LC110353 | 1. 504 | 1. mouse | 1. *Mus musculus* | 1. 1 | 1. JPN | | 1. - | 1. - | 1. 723 | 1. 241 | 1. 1731 | 1. 577 | 1. 1281 | 1. 427 |
| 1. KY855431 | 1. HT4 | 1. marmot | 1. *Marmota himalayana* | 1. 1 | 1. CHN | | 1. - | 1. - | 1. 570 | 1. 190 | 1. 1713 | 1. 571 | 1. 1590 | 1. 530 |
| 1. KU729753; KU729767 | 1. PF090307 | 1. otarine | 1. *Zalophus californianus* | 1. 1 | 1. CHN | | 1. - | 1. - | 1. 669 | 1. 223 | 1. 1668 | 1. 556 | 1. 1587 | 1. 529 |
| 1. KJ663813; KJ663814 | 1. CDC23 | 1. human | 1. *Homo sapiens* | 1. 2 | 1. USA | | 1. 120 | 1. 40 | 1. 396 (a), 2. 348 (b) | 1. 132 (a), 2. 116 (b) | 1. 1656 | 1. 552 | 1. 1596 | 1. 532 |
| 1. KR902504; KR902503 | 1. Equ1 | 1. horse | 1. *Equus caballus* | 1. 2 | 1. USA | | 1. - | 1. - | 1. 453 | 1. 151 | 1. 1581 | 1. 527 | 1. 1584 | 1. 528 |
| 1. KY855429 | 1. HT2 | 1. marmot | 1. *Marmota himalayana* | 1. 2 | 1. CHN | | 1. - | 1. - | 1. 933 | 1. 311 | 1. 1638 | 1. 546 | 1. 1638 | 1. 546 |
| 1. AF246940 | 1. 4GA91 | 1. human | 1. *Homo sapiens* | 1. 2 | 1. USA | | 1. n.s | 1. n.s | 1. n.s | 1. n.s | 1. n.s | 1. n.s | 1. 1551 | 1. 517 |
| 1. AP014890; AP014891 | 1. PBV | 1. diatom |  | 1. 3 | 1. JPN | | 1. - | 1. - | 1. - | 1. - | 1. 1521 | 1. 507 | 1. 1515 | 1. 505 |
| 1. KX884078 | 1. BHZC36678 | 1. razor shell | 1. *Solenidae* | 1. 3 | 1. CHN | | 1. - | 1. - | 1. - | 1. - | 1. 1686 | 1. 562 | 1. 1545 | 1. 515 |

*without stop codon; aa, number of amino acids; nt, number of nucleotides; RdRP, RNA-dependent RNA polymerase; DEU, Germany; THA, Thailand; JPN, Japan; CHN, China; USA, United States of America

References

1. Wilharm, G. *et al.* Relatedness of wildlife and livestock avian isolates of the nosocomial pathogen *Acinetobacter baumannii* to lineages spread in hospitals worldwide. *Environ Microbiol.* **19,** 4349–4364 (2017).

2. Mrochen, D. M., Fernandes de Oliveira, L. M., Raafat, D. & Holtfreter, S. *Staphylococcus aureus* Host Tropism and Its Implications for Murine Infection Models. *Int J Mol Sci.* **21** (2020).

3. Bader, O. *et al.* Improved clinical laboratory identification of human pathogenic yeasts by matrix-assisted laser desorption ionization time-of-flight mass spectrometry. *Clin Microbiol Infect.* **17,** 1359–1365 (2011).

4. Sauer, S. *et al.* Classification and identification of bacteria by mass spectrometry and computational analysis. *PloS One.* **3,** e2843 (2008).

5. Fenselau, C. & Demirev, P. A. Characterization of intact microorganisms by MALDI mass spectrometry. *Mass Spectrom Rev.* **20,** 157–171 (2001).

6. Krishnamurthy, T. & Ross, P. L. Rapid Identification of Bacteria by Direct Matrix-assisted Laser Desorption/Ionization Mass Spectrometric Analysis of Whole Cells. *Rapid Commun Mass Spectrom.* **10,** 1992–1996 (1996).

7. Holland, R. D. *et al.* Rapid Identification of Intact Whole Bacteria Based on Spectral Patterns using Matrix-assisted Laser Desorption/Ionization with Time-of-flight Mass Spectrometry. *Rapid Commun Mass Spectrom.* **10,** 1227–1232 (1996).

8. Wylezich, C., Papa, A., Beer, M. & Höper, D. A Versatile Sample Processing Workflow for Metagenomic Pathogen Detection. *Sci Rep.* **8,** 13108 (2018).

9. Schmidt, K. *et al.* Development of a multiplex serological assay reveals a worldwide distribution of murine astrovirus infections in laboratory mice. *PloS One.* **12,** e0187174 (2017).

10. Waterboer, T. *et al.* Multiplex human papillomavirus serology based on in situ-purified glutathione s-transferase fusion proteins. *Clin Chem.* **51,** 1845–1853 (2005).

11. Mayer-Scholl, A. *et al. Leptospira* spp. in rodents and shrews in Germany. *Int J Environ Res Public Health.* **11,** 7562–7574 (2014).

12. Ehlers, B., Richter, D., Matuschka, F.-R. & Ulrich, R. G. Genome Sequences of a Rat Polyomavirus Related to Murine Polyomavirus, Rattus norvegicus Polyomavirus 1. *Genome Announc.* **3** (2015).

13. Jahfari, S. *et al.* Prevalence of *Neoehrlichia mikurensis* in ticks and rodents from North-west Europe. *Parasit Vectors.* **5,** 74 (2012).

14. Maggi, R. G., et al. The use of molecular diagnostic techniques to detect *Anaplasma*, *Bartonella* and *Ehrlichia* species in arthropods or patients, “The international canine vector-borne disease symposium, April 18^th^-20^th^, 2006.” Billesley (2006): 9-14.

15. Courtney, J. W., Kostelnik, L. M., Zeidner, N. S. & Massung, R. F. Multiplex real-time PCR for detection of *Anaplasma phagocytophilum* and *Borrelia burgdorferi*. *J Clin Microbiol.* **42,** 3164–3168 (2004).

16. Casati, S., Sager, H., Gern, L. & Piffaretti, J.-C. Presence of potentially pathogenic *Babesia* sp. for human in *Ixodes ricinus* in Switzerland. *Ann Agric Environ Med. AAEM* **13,** 65–70 (2006).

17. Klempa, B. *et al.* Hantavirus in African wood mouse, Guinea. *Emerg Infect Dis.* **12,** 838–840 (2006).

18. Essbauer, S. *et al.* A new Puumala hantavirus subtype in rodents associated with an outbreak of Nephropathia epidemica in South-East Germany in 2004. *Epidemiol Infect.* **134,** 1333–1344 (2006).

19. Hoffmann et al., In-house assay, unpublished.

20. Scaramozzino, N. *et al.* Real-time PCR to identify variola virus or other human pathogenic orthopox viruses. *Clin Chem.* **53,** 606–613 (2007).

21. Johne, R. *et al.* Detection of a novel hepatitis E-like virus in faeces of wild rats using a nested broad-spectrum RT-PCR. *J Gen Virol.* **91,** 750–758 (2010).

22. Schulz, E. *et al.* Genomic characterization of the first insectivoran papillomavirus reveals an unusually long, second non-coding region and indicates a close relationship to Betapapillomavirus. *J Gen Virol.* **90,** 626–633 (2009).

23. Schwaiger, M., Péter, O. & Cassinotti, P. Routine diagnosis of *Borrelia burgdorferi* (sensu lato) infections using a real-time PCR assay. *Clin Microbiol Infect.* **7,** 461–469 (2001).

24. Wölfel, R., Essbauer, S. & Dobler, G. Diagnostics of tick-borne rickettsioses in Germany: A modern concept for a neglected disease. *Int J Med Microbiol.* **298,** 368–374 (2008).

25. Jacobsen, S. *et al.* Co-circulation of classic and novel astrovirus strains in patients with acute gastroenteritis in Germany. *J Infect.* **76,** 457–464 (2018).

26. In-house assay, unpublished.

27. Kuryk, Ł. *et al.* Genetic analysis of poliovirus strains isolated from sewage in Poland. *J Med Virol.* **86,** 1243–1248 (2014).

28. Adlhoch, C. *et al.* Highly sensitive detection of the group A rotavirus using apolipoprotein H-coated ELISA plates compared to quantitative real-time PCR. *Virol J.* **8,** 63 (2011).

29. Altona RealStar Norovirus PCR Kit. https://www.diagnostictechnology.com.au/products/altona-real-star-norovirus-rt-pcr-kit-1-0; Access 03.05.2017

30. Altona RealStar Adenovirus PCR Kit. https://www.diagnostictechnology.com.au/products/altona-realstar-adenovirus-pcr-kit-1-0; Access 27.04.2017

31. Altona RealStar Clostridium difficile PCR Kit. https://www.diagnostictechnology.com.au/products/altona-realstar-clostridium-difficile-pcr-kit-1-0; Access 15.05.2017

32. Fawzy, A. *et al.* Development and validation of a triplex real-time qPCR for sensitive detection and quantification of major rat bite fever pathogen *Streptobacillus moniliformis*. *J Microbiol Methods.* **199,** 106525 (2022).

33. Lanciotti, R. S. *et al.* Genetic and serologic properties of Zika virus associated with an epidemic, Yap State, Micronesia, 2007. *Emerg Infect Dis.* **14,** 1232–1239 (2008).

34. Jöst, H. *et al.* Isolation and phylogenetic analysis of Sindbis viruses from mosquitoes in Germany. *J Clin Microbiol.* **48,** 1900–1903 (2010).

35. Panning, M., Grywna, K., van Esbroeck, M., Emmerich, P. & Drosten, C. Chikungunya fever in travelers returning to Europe from the Indian Ocean region, 2006. *Emerg Infect Dis.* **14,** 416–422 (2008).

36. Vina-Rodriguez, A., Eiden, M., Keller, M., Hinrichs, W. & Groschup, M. H. A Quantitative Real-Time RT-PCR Assay for the Detection of Venezuelan equine encephalitis virus Utilizing a Universal Alphavirus Control RNA. *Biomed Res Int.* **2016,** 8543204 (2016).

37. Lambert, A. J., Martin, D. A. & Lanciotti, R. S. Detection of North American eastern and western equine encephalitis viruses by nucleic acid amplification assays. *J Clin Microbiol.* **41,** 379–385 (2003).

38. Vieth, S. *et al.* RT-PCR assay for detection of Lassa virus and related Old World arenaviruses targeting the L gene. *Trans R Soc Trop Med Hyg.* **101,** 1253–1264 (2007).

39. Vieth, S., Drosten, C., Charrel, R., Feldmann, H. & Günther, S. Establishment of conventional and fluorescence resonance energy transfer-based real-time PCR assays for detection of pathogenic New World arenaviruses. *J Clin Virol.* **32,** 229–235 (2005).

40. In-house ELISA, unpublished.

41. AXIOM HEV-Ab ELISA Kit, AXIOM Diagnostic www.axiom-solutions.de; Access: 23.04.2017.

42. Heuser, E. *et al.* Pet Rats as the Likely Reservoir for Human Seoul Orthohantavirus Infection. *Viruses* **15** (2023).
